# HUBO and QUBO models for prime factorization

**DOI:** 10.1038/s41598-023-36813-x

**Published:** 2023-06-21

**Authors:** Kyungtaek Jun, Hyunju Lee

**Affiliations:** 1 Research center, Qtomo, Busan, South Korea; 2 Institute of Mathematical Sciences, Ewha Womans University, Seoul, South Korea; 3 German Engineering Research and Development Center, LSTME Busan Branch, Busan, South Korea; 4 University-Industry Foundation, Yonsei University Health System, Seoul, South Korea

**Keywords:** Applied mathematics, Computational science

## Abstract

The security of the RSA cryptosystem is based on the difficulty of factoring a large number N into prime numbers $p$ and $q$ satisfying $N=p\times q$. This paper presents a prime factorization method using a D-Wave quantum computer that could threaten the RSA cryptosystem in the future. The starting point for this method is very simple, representing two prime numbers as qubits. Then, we set the difference between the product of the two prime numbers expressed in qubits and N as a cost function, and we find the solution when the cost function is minimized. D-Wave's quantum annealer can find the minimum value of any quadratic problem. However, the cost function must be a higher-order unconstrained optimization (HUBO) model because it contains second- or higher-order terms. We used a hybrid solver accessible via Leap, D-Wave’s real-time quantum cloud service, and the *dimod* package provided by the D-Wave Ocean software development kit (SDK) to solve the HUBO problem. We also successfully factorized 102,454,763 with 26 logical qubits. In addition, we factorized 1,000,070,001,221 using the range-dependent Hamiltonian algorithm.

## Introduction

The RSA cryptosystem is a popular public-key cryptographic algorithm for secure data transmission that was first introduced in 1978^1^. Public and private keys form a pair in the RSA cryptosystem. Content encrypted with the public key can only be decrypted with the private key, and content encrypted with the private key can only be decrypted with the public key. The public key is a large biprime that can only be decrypted through prime factors held in the private key. The RSA cryptosystem is based on prime factorization^2^, which finds the prime factors $p$ and $q$ such that $N=p\times q$ for a large biprime $N$. This algorithm is an interesting mathematical problem because it relies on principles of number theory. While the RSA cryptosystem is surprisingly simple, a large biprime is difficult to factorize^3^. In other words, the computational complexity of decryption is much higher than that of encryption, so security is excellent. However, with the development of quantum computers, quantum algorithms that reduce the computational complexity difference between encryption and decryption have been proposed, threatening the safety of the RSA cryptosystem.

Peter Shor proposed the Shor algorithm^4^, a powerful quantum algorithm that can factorize an integer N in polynomial time and can therefore attack the RSA cryptosystem in polynomial time, in 1994^5^. There have been many simulations on quantum computers for practical use—moreover, many attempts have been made to run the Shor algorithm on quantum computer hardware. Geller et al. applied the Shor algorithm to factorize 51 and 85 using Fermat numbers and eight qubits^6^. However, there are still limitations to applying Shor's algorithm to a large number. On the other hand, quantum annealing, which underlies D-Wave quantum computers, allows the factorization of even larger numbers. D-Wave quantum annealing can find the minimum value of a quadratic unconstrained binary optimization (QUBO) or Ising model^7^. The objective, formulated as the problem Hamiltonian of a D-Wave quantum computer, is achieved by defining the linear and quadratic coefficients of a binary quadratic model (BQM) that maps those values to the qubits and couplers of the QPU. Dridi et al. presented a new autonomous algorithm that factorizes all biprimes up to 200,099 by reducing the Hamiltonian's degree using Gröbner bases using the D-Wave 2X processor with more than 1000 qubits^8^. However, the Gröbner basis calculation is exponential in the number of variables, so factorizing larger numbers can require significantly more qubits. Jiang et al. developed a frame that transforms a prime factorization problem for arbitrary integers into an Ising model using ancillary variables and presented a way to factorize the integer 376,289 with 94 logical qubits via a D-Wave 2000Q System^9^. However, it also faces the limitations of the hardware of the quantum computer. Due to the limitation of the number of usable qubits of the D-Wave 2000Q system, many researchers have studied the factorization of larger integers using the D-Wave hybrid quantum/classical simulator *qbsolv*. Peng et al. improved on Jiang et al.’s work and factored 1,005,973 with 89 qubits by using *qbsolv*^10^. Wang et al. successfully factorized all integers less than or equal to 10,000 and factorized 1,028,171 with 88 qubits via *qbsolv*^11^. Additionally, Wang et al. recently factorized 1,630,729 (an 11-bit prime factor multiplied by an 11-bit prime factor)^12^. Unfortunately, a generalized method for factoring all integers into primes has not been developed due to the limitations of current quantum computers.

Jiang et al. proposed a direct method for prime factorization. In this paper, we propose a direct method to formulate a higher-order unconstrained optimization (HUBO) model for prime factorization. To obtain the HUBO model, it is sufficient to express *p* and *q*, which are prime factors of biprime *N*, as sums of qubits. We used a solver accessible via Leap™, D-Wave's cloud-based platform, which provides real-time access to a D-Wave quantum computer, to obtain the minimum value of the HUBO model^13^. In Leap™, there are QPU solvers accessible to D-Wave 2000Q and Advantage quantum computers and hybrid solvers that use both classical and quantum resources. The numbers of usable qubits for the D-wave 2000Q and Advantage QPUs are more than 2000 and 5000, respectively. The hybrid solver is designed to accommodate even very large problems and is an updated version of *qbsolv*. Therefore, we used the Advantage QPU solver to prime factorize $N = 15$ in the Advantage quantum computer and used a hybrid solver to factorize much larger numbers. In addition, the hybrid solver can solve arbitrary problems formulated as quadratic models. Therefore, a process of converting the HUBO model to a QUBO model is required to solve a problem using a hybrid solver. The composed sampler in the *dimod* package of the Ocean software development kit (SDK), a set of open-source Python Tools provided by D-Wave Systems, creates a BQM from a higher-order problem. Hence, if these tools are used, the HUBO model is converted into a QUBO model, and then the prime factorization of large integers is possible using the D-Wave Ocean SDK. We demonstrated the factorization of 102,454,763 with 26 logical qubits using the D-Wave hybrid solver as a successful example of our method. Due to the limitations of the numerical values that the hybrid solver can use in the quantum annealer for the coefficients of the QUBO model, numbers much larger than the previous number are difficult to factor with our new HUBO method. To address this limitation, we applied a range-dependent Hamiltonian algorithm that divides the domain to the new HUBO model. This algorithm is a method of finding a solution with a small number of qubits by dividing the domain into certain subintervals. As a result, the number 1,000,070,001,221 was prime factorized using a HUBO model to which this algorithm was applied. For not only this number but also numbers smaller or larger than this, if the QUBO/HUBO model obtained by our methods has fewer qubits than the number of qubits available in the hybrid solver and the model's coefficients are accurately expressed, prime factorization is possible. Thus, more numbers will be prime factorized in the future as the number of available qubits in the hybrid solver increases and the coefficients are accurately represented.

## Methods

### The least-squares problem for prime factorization

The Ising model is a mathematical model for ferromagnetism in statistical mechanics. The energy Hamiltonian (the cost function) is formulated as follows:

1 $$H\left(\overrightarrow{\sigma }\right)=-\sum_{i=1}^{n}{h}_{i}{\sigma }_{i}-\sum_{i<j}^{n}{J}_{i,j}{\sigma }_{i}{\sigma }_{j}$$

where $\overrightarrow{\sigma }={\left({\sigma }_{1},\cdots ,{\sigma }_{n}\right)}^{T}$ and ${\sigma }_{i}\in \{+1,-1\}$. ${\sigma }_{i}$ represents the qubit (quantum bit) spin, and ${h}_{i}$ and ${J}_{i,j}$ are the coefficients for the qubit spins and couplers, respectively^14^.

QUBO is a combinatorial optimization problem in computer science. In this problem, a cost function *f* is defined on an $n-$ dimensional binary vector space ${\mathbb{B}}^{n}$ onto ${\mathbb{R}}$.

2 $$f\left(\overrightarrow{q}\right)={\overrightarrow{q}}^{T}Q\overrightarrow{q}$$

where *Q* is an upper diagonal matrix, $\overrightarrow{q}={\left({q}_{1},\cdots ,{q}_{n}\right)}^{T}$, and ${q}_{i}$ is a binary element of $\overrightarrow{q}$. In this paper, the matrix Q is referred to as the QUBO matrix. The problem is to find $\overrightarrow{{q}^{*}}$ that minimizes the cost function $f$ among vectors $\overrightarrow{q}$. Since we have ${q}_{i}^{2}={q}_{i}$, the cost function is reformulated as follows:

3 $$f\left(\overrightarrow{x}\right)=\sum_{i=1}^{n}{Q}_{i,i}{q}_{i}+\sum_{i<j}^{n}{Q}_{i,j}{q}_{i}{q}_{j}$$

In *Q*, the diagonal terms ${Q}_{i,i}$ and off-diagonal terms ${Q}_{i,j}$ represent the linear terms and quadratic terms, respectively. The unknowns of the Ising model *σ* and the unknowns of the QUBO model *q* have the linear relation

4 $$\sigma \to 2q-1 \mathrm{or} q\to \frac{1}{2}(\sigma +1)$$

Assume that the integer $N$ is the product of two prime numbers $p$ and $q$. To calculate $p$ and $q$, consider the following least-squares problem:

5 $$\underset{{p,q}}{\text{arg min}}\parallel {pq-N}\parallel$$

Equation (1) reaches the minimum value $0$ when $pq=N$. To compute Eq. (5) conveniently, we apply the 2-norm square to it.

6 $$\parallel pq-N{\parallel }_{2}^{2}={p}^{2}{q}^{2}-2pqN+N^{2}$$

### HUBO model

When solving a binary least-squares problem, $p$ and $q$ are represented by combinations of qubits ${q}_{l}\in \{\mathrm{0,1}\}$. The radix 2 representation of the positive integer values $p$ and $q$ is given by

7 $$p\approx \sum_{l=0}^{n-1}{2}^{l}{q}_{l} \;\; \mathrm{and} \;\;q\approx \sum_{l=0}^{n-1}{2}^{l}{q}_{n+l}$$

where the integer $l+1$ denotes the number of binary digits of $p$ and $q$^15^. We use qubits from $l = 0$ to use the same equation in the range-dependent Hamiltonian algorithm in Chapter 2.4.

To derive a HUBO model, we insert Eq. (7) into Eq. (6). This yields the summation terms of the first term in Eq. (6), as indicated below:

8 $${p}^{2}{q}^{2}={\left(\sum_{l=0}^{n-1}{2}^{l}{q}_{l}\right)}^{2}{\left(\sum_{l=0}^{n-1}{2}^{l}{q}_{n+l}\right)}^{2}$$

9 $$=\left(\sum_{l=0}^{n-1}{2}^{2l}{q}_{l}+\sum_{{l}_{1}<{l}_{2}}{2}^{{l}_{1}+{l}_{2}+1}{q}_{{l}_{1}}{q}_{{l}_{2}}\right)\left(\sum_{l=0}^{l-1}{2}^{2l}{q}_{n+l}+\sum_{{l}_{1}<{\mathrm{l}}_{2}}{2}^{{l}_{1}+{l}_{2}+1}{q}_{n+{l}_{1}}{q}_{n+{l}_{2}}\right)$$

10 $$\begin{aligned} &= \mathop \sum \limits_{{l_{1} = 0}}^{n - 1} \mathop \sum \limits_{{l_{2} = 0}}^{n - 1} 2^{{2(l_{1} + l_{2} )}} q_{{l_{1} }} q_{{n + l_{2} }} + \mathop \sum \limits_{{l_{1} = 0}}^{n - 1} \mathop \sum \limits_{{l_{2} < l_{3} }} 2^{{2l_{1} + l_{2} + l_{3} + 1}} \left( {q_{{l_{1} }} q_{{n + l_{2} }} q_{{n + l_{3} }} + q_{{l_{2} }} q_{{l_{3} }} q_{{n + l_{1} }} } \right) \hfill \\ & \quad + \mathop \sum \limits_{{l_{1} < l_{2} }} \mathop \sum \limits_{{l_{3} < l_{4} }} 2^{{l_{1} + l_{2} + l_{3} + l_{4} + 2}} q_{{l_{1} }} q_{{l_{2} }} q_{{n + l_{3} }} q_{{n + l_{4} }} \hfill \\ \end{aligned}$$

In Eq. (8), since ${\left({q}_{l}\right)}^{2}={q}_{l}$, Eq. (9) can be obtained. The second term of Eq. (6) is calculated as follows:

11 $$-2pqN=-2N\left(\sum_{l=0}^{n-1}{2}^{l}{q}_{l}\right)\left(\sum_{l=0}^{n-1}{2}^{l}{q}_{n+l}\right)$$

12 $$=\sum_{{l}_{1}=0}^{n-1}\sum_{{l}_{2}=0}^{n-1}\left(-{2}^{{l}_{1}+{l}_{2}+1}{Nq}_{{l}_{1}}{q}_{n+{l}_{2}}\right)$$

The HUBO model consists of the sum of Eqs. (10) and (12). The global minimum energy we need to obtain is $-{N}^{2}$.

### QUBO model

The HUBO model for prime factorization consists of quadratic, cubic, and quartic terms. To reformulate a nonquadratic (higher-degree) polynomial into QUBO form, terms of the form $cxyz$, where *c* is a real number, are substituted with one of the following quadratic terms^9^:

13 $$cxyz=\left\{\begin{array}{ll}cw\left(x+y+z-2\right), & \quad c<0\\ c\left\{w\left(x+y+z-1\right)+\left(xy+yz+zx\right)-\left(x+y+z\right)+1\right\}, & \quad c>0\end{array}\right.$$

For all $x,y,z\in \{\mathrm{0,1}\}$, $cxyz$ can be transformed into a combination of linear and quadratic terms by adding a new qubit *w* to every cubic term. Similarly, Eq. (13) can be applied twice to convert quartic terms into QUBO formulations. The quartic terms with positive coefficients in this HUBO model are calculated as follows:

14 $${a}_{1}{a}_{2}{b}_{1}{b}_{2}={a}_{1}\left({x}_{1}{a}_{2}+{x}_{1}{b}_{1}+{x}_{1}{b}_{2}+{a}_{2}{b}_{1}+{a}_{2}{b}_{2}+{b}_{1}{b}_{2}-{a}_{2}-{b}_{1}-{b}_{2}-{x}_{1}+1\right)$$

15 $$\begin{aligned} & = x_{2} x_{1} + x_{2} a_{1} + x_{2} a_{2} + x_{1} a_{1} + x_{1} a_{2} + a_{1} a_{2} - x_{1} - a_{1} - a_{2} - x_{2} + 1 \hfill \\ & \quad + x_{3} x_{1} + x_{3} a_{1} + x_{3} b_{1} + x_{1} a_{1} + x_{1} b_{1} + a_{1} b_{1} - x_{1} - a_{1} - b_{1} - x_{3} + 1 \hfill \\ & \quad + x_{4} x_{1} + x_{4} a_{1} + x_{4} b_{2} + x_{1} a_{1} + x_{1} b_{2} + a_{1} b_{2} - x_{1} - a_{1} - b_{2} - x_{4} + 1 \hfill \\ & \quad + x_{5} a_{1} + x_{5} a_{2} + x_{5} b_{1} + a_{1} a_{2} + a_{1} b_{1} + a_{2} b_{1} - a_{1} - a_{2} - b_{1} - x_{5} + 1 \hfill \\ & \quad + x_{6} a_{1} + x_{6} a_{2} + x_{6} b_{2} + a_{1} a_{2} + a_{1} b_{2} + a_{2} b_{2} - a_{1} - a_{2} - b_{2} - x_{6} + 1 \hfill \\ & \quad + x_{7} a_{1} + x_{7} b_{1} + x_{7} b_{2} + a_{1} b_{1} + a_{1} b_{2} + b_{1} b_{2} - a_{1} - b_{1} - b_{2} - x_{7} + 1 \hfill \\ & \quad - a_{1} a_{2} - a_{1} b_{1} - a_{1} b_{2} - a_{1} x_{1} + a_{1} \hfill \\ \end{aligned}$$

For all ${a}_{1},{a}_{2},{b}_{1},{b}_{2},\in \{\mathrm{0,1}\}$, ${a}_{1}{a}_{2}{b}_{1}{b}_{2}$ can be transformed into a combination of linear and quadratic terms by adding seven new qubits, ${x}_{1}, {x}_{2},\cdots ,{x}_{7}$, for each quartic term.

### HUBO model with the range-dependent Hamiltonian algorithm

Recently, the range-dependent Hamiltonian algorithm was proposed^16^. This algorithm divides the domain into subregions that can be represented by the desired number of qubits. Applying this algorithm, $p$ and $q$ can be expressed as follows:

16 $$p\approx \sum_{l=0}^{n-1}{2}^{l}{q}_{l}+{S}_{i}\;\; \mathrm{and} \;\; q \approx \sum_{l=0}^{n-1}{2}^{l}{q}_{n+l}+{S}_{j}$$

where ${S}_{k}=k{2}^{n}$ and $k$ is an integer.

To derive a HUBO model, we insert Eq. (16) into Eq. (6). This yields the summation terms of the first term in Eq. (6), as indicated below:

17 $${p}^{2}{q}^{2}-{S}_{i}^{2}{S}_{j}^{2}={\left(\sum_{l=0}^{n-1}{2}^{l}{q}_{l}+{S}_{i}\right)}^{2}{\left(\sum_{l=0}^{n-1}{2}^{l}{q}_{n+l}+{S}_{j}\right)}^{2}-{S}_{i}^{2}{S}_{j}^{2}$$

18 $$=\left(\sum_{l=0}^{n-1}\left({2}^{2l}+{2}^{l+1}{S}_{i}\right){q}_{l}+\sum_{{l}_{1}<{l}_{2}}{2}^{{l}_{1}+{l}_{2}+1}{q}_{{l}_{1}}{q}_{{l}_{2}}+{S}_{i}^{2}\right)\left(\sum_{l=0}^{n-1}\left({2}^{2l}+{2}^{l+1}{S}_{j}\right){q}_{n+l}\left.+\sum_{{l}_{1}<{l}_{2}}{2}^{{l}_{1}+{l}_{2}+1}{q}_{n+{l}_{1}}{q}_{n+{l}_{2}}+{S}_{j}^{2}\right)-{S}_{i}^{2}{S}_{j}^{2}\right.$$

19 $$\begin{aligned} &= \mathop \sum \limits_{l = 0}^{n - 1} \left( {\left( {2^{2l} + 2^{l + 1} S_{i} } \right)S_{j}^{2} q_{l} + \left( {2^{2l} + 2^{l + 1} S_{j} } \right)S_{i}^{2} q_{n + l} } \right) + \mathop \sum \limits_{l = 0}^{n - 1} \left( {2^{{l_{1} + l_{2} + 1}} S_{j}^{2} q_{{l_{1} }} q_{{l_{2} }} + 2^{{l_{1} + l_{2} + 1}} S_{i}^{2} q_{{n + l_{1} }} q_{{n + l_{2} }} } \right) \hfill \\ & \quad + \mathop \sum \limits_{{l_{1} = 0}}^{n - 1} \mathop \sum \limits_{{l_{2} = 0}}^{n - 1} \left( {2^{{2(l_{1} + l_{2} )}} + 2^{{l_{1} + 2l_{2} + 1}} S_{i} + 2^{{2l_{1} + l_{2} + 1}} S_{j} + 2^{{l_{1} + l_{2} + 2}} S_{i} S_{j} } \right)q_{{l_{1} }} q_{{n + l_{2} }} \hfill \\ & \quad + \mathop \sum \limits_{{l_{1} = 0}}^{n - 1} \mathop \sum \limits_{{l_{2} < l_{3} }} \left( {2^{{l_{2} + l_{3} + 1}} \left( {2^{{2l_{1} }} + 2^{{l_{1} + 1}} S_{j} } \right)} \right)q_{{l_{2} }} q_{{l_{3} }} q_{{n + l_{1} }} + \mathop \sum \limits_{{l_{1} = 0}}^{n - 1} \mathop \sum \limits_{{l_{2} < l_{3} }} \left( {2^{{l_{2} + l_{3} + 1}} \left( {2^{{2l_{1} }} + 2^{{l_{1} + 1}} S_{i} } \right)} \right)q_{{l_{1} }} q_{{n + l_{2} }} q_{{n + l_{3} }} \hfill \\ & \quad + \mathop \sum \limits_{{l_{1} < l_{2} }} \mathop \sum \limits_{{l_{3} < l_{4} }} 2^{{l_{1} + l_{2} + l_{3} + l_{4} + 2}} q_{{l_{1} }} q_{{l_{2} }} q_{{n + l_{3} }} q_{{n + l_{4} }} \hfill \\ \end{aligned}$$

In Eq. (8), since ${\left({q}_{l}\right)}^{2}={q}_{l}$, Eq. (9) can be obtained. The second term of Eq. (6) is calculated as follows:

20 $$-2N\left(pq-{S}_{i}{S}_{j}\right)=-2N\left(\sum_{l=0}^{n-1}{2}^{l}{q}_{l}+{S}_{i}\right)\left(\sum_{l=0}^{n-1}{2}^{l}{q}_{n+l}+{S}_{j}\right)+2{NS}_{i}{S}_{j}$$

21 $$=-\sum_{l=0}^{n-1}{2}^{l+1}N\left({S}_{j}{q}_{l}+{S}_{i}{q}_{n+l}\right)-\sum_{{l}_{1}=0}^{n-1}\sum_{{l}_{2}=0}^{n-1}{2}^{{l}_{1}+{l}_{2}+1}{Nq}_{{l}_{1}}{q}_{n+{l}_{2}}$$

The HUBO model consists of the sum of Eqs. (19) and (21). The global minimum energy we need to obtain is $-{N}^{2}-{S}_{i}^{2}{S}_{j}^{2}+2{NS}_{i}{S}_{j}$.

## Experiments

We use a hybrid solver that calculates the global minimum energy using a combination of a quantum annealer and a classical computer, dimod.ExactPolySolver().sample_hubo(), in the D-Wave Ocean SDK to test the HUBO model for the prime factorizations. This solver represents the energy for every possible number of cases each qubit can have. For evaluating this algorithm, we randomly selected 100 different numbers among the first thousand prime numbers and tested it on the selected numbers. In all cases, we were able to find pairs $pq$ and $qp$ using the new HUBO model. The largest number we tested was 102,454,763. The solver factored the number into two prime numbers, 10,111 and 10,133. The pseudocode used in the test is shown in Algorithm 1.
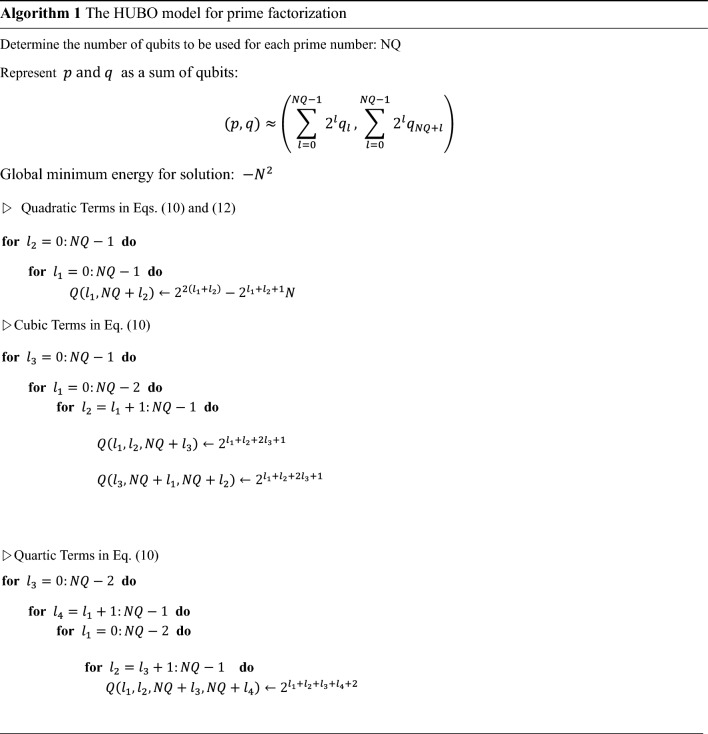


To calculate the first quadratic terms, we multiply ${2}^{{l}_{1}}$ and ${2}^{{l}_{2}}$ and then square them for ${n}^{2}$ loops. The total number of flops is $2({n+1)}^{2}$. In a similar way, a total of $3{(n+1)}^{2}$ flops can be obtained for the second quadratic terms. The total number of flops for the quadratic terms is $5{(n+1)}^{2}$. We can consider the change in terms of the first quadratic terms. ${2}^{2\left({l}_{1}+{l}_{2}\right)}$ varies from ${4}^{0}$ to ${4}^{2n-2}$. We can represent all of these terms as a sum of $2n+1$. Rather than directly calculating each coefficient, if we find the amount of change and put it into the coefficient appropriately, we can reduce the amount of calculation by approximately the square root. In a similar way, calculating the change amounts for the cubic term and the quartic term requires $3n+1$ and $4n+1$ flops, respectively. We can reduce the amount of calculation once more here. The coefficients of the first quadratic terms and cubic terms are included in the coefficients of the quartic terms. Therefore, the total optimized number of flops for the HUBO model is $(4n+3)+(2n+3)$.

Both the cubic and quartic terms of this HUBO model have positive coefficients. Therefore, the QUBO model for the factorization can be formulated using Eqs. (13) and (15). The pseudocode is shown in Algorithm 2. In Algorithm 2, the cubic terms are divided into linear terms, quadratic terms, and constant terms by Eq. (13). The constant term generated here is not included in the QUBO model and appears in the reduced form of the global minimum energy.
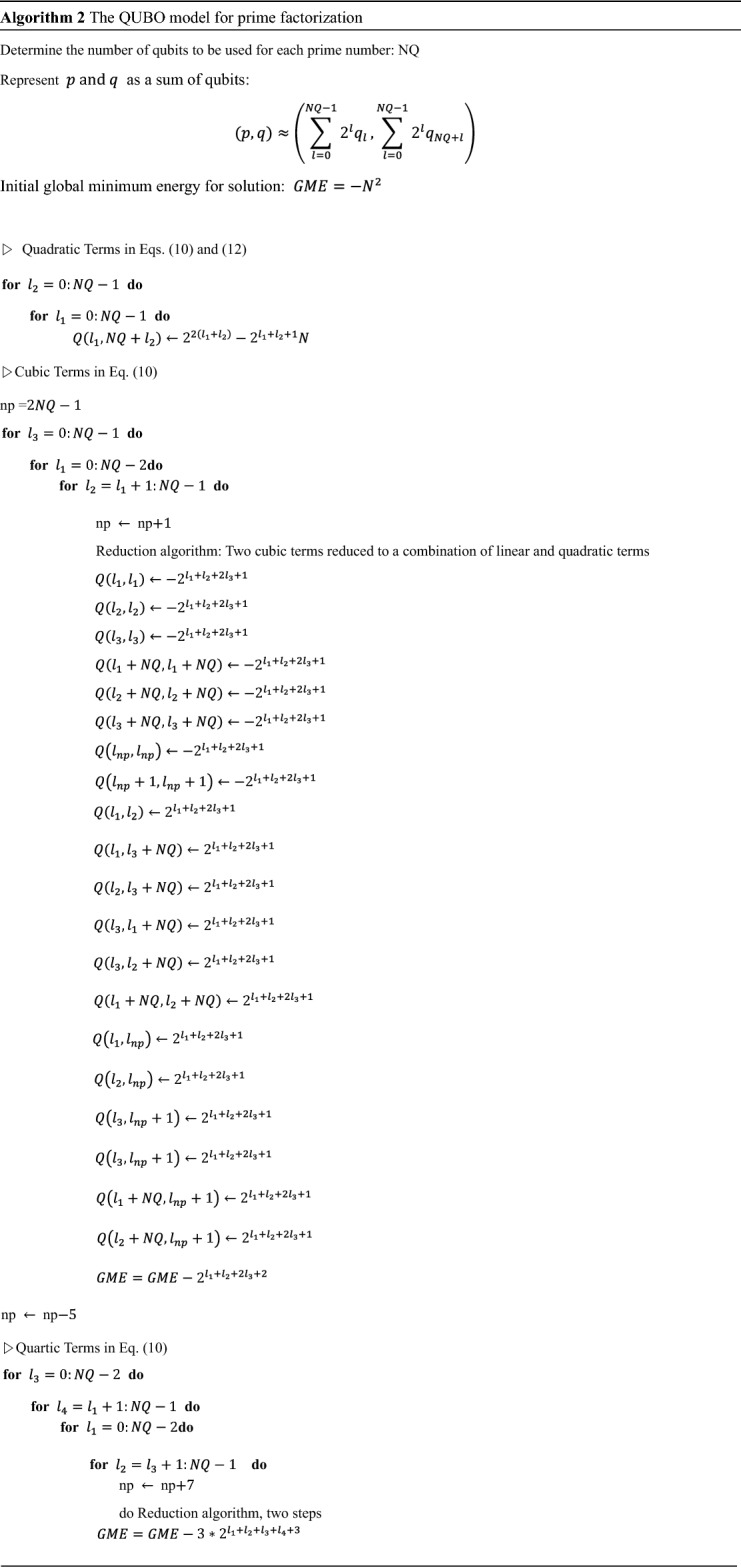


Two cubic terms with positive coefficients can be formulated into 10 linear and quadratic terms using Eq. (13). In the process of converting the two cubic terms into the form of a QUBO model, two new qubits are added. Equation (15) is used to formulate each quartic term in the form of a QUBO model. First, Eq. (13) is applied to three qubits. Then, the remaining qubit is multiplied by the newly created terms. Here, Eq. (15) is formulated by applying Eq. (13) again to the newly created cubic terms. By formulating cubic and quartic terms as linear and quadratic terms, the QUBO model for prime factorization can be obtained.

**Figure 1:**
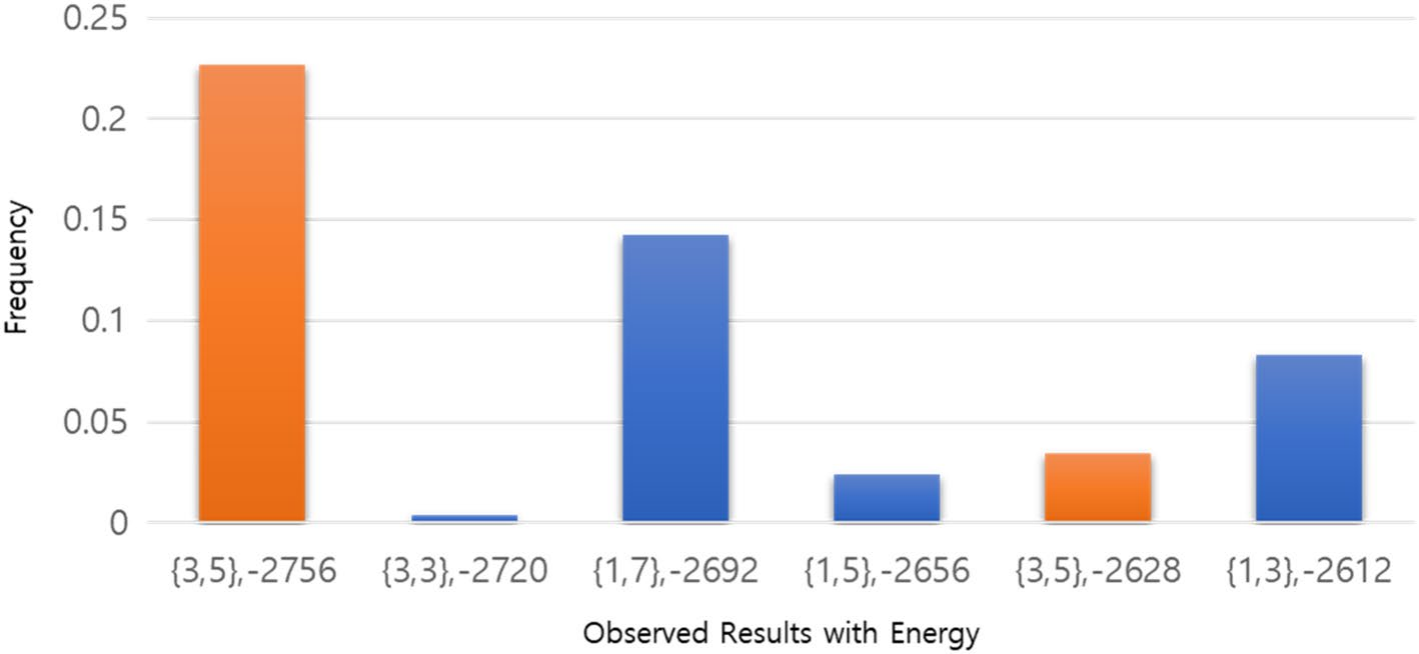
Experimental results of the QUBO model on the D-Wave machine to factorize $N=15$. The x-axis represents the solution and energy, and the y-axis represents the frequency. The solution was obtained with a QPU solver using 15 logical qubits. Orange bars represent cases where the pair of $p$ and $q$ is the solution {3,5}, blue bars represent cases where they are not.

We prime factorize the biprime number $N$ by two prime numbers, $p$ and $q$, to test the QUBO model for prime factorization. Figure 1 is the result of $N=15$ obtained using a QPU solver for the QUBO model. In the process of calculating the HUBO model as a QUBO model, different energies appear for the same solution because new logical qubits are used. The energy we can see as a solution is the global minimum energy of − 2756.

Finally, we apply the range-dependent algorithm to the HUBO model to factorize 1,000,070,001,221. The pseudocode used here can be seen in Algorithm 3. The HUBO model to which this algorithm is applied shows the global minimum energy $-\mathrm{4,900,170,941,490,841}$ only in the section where the solution exists. To solve the HUBO model with a range-dependent algorithm, we used 12 logical qubits, and each ${S}_{i}$ and ${S}_{j}$ is 1,000,000.
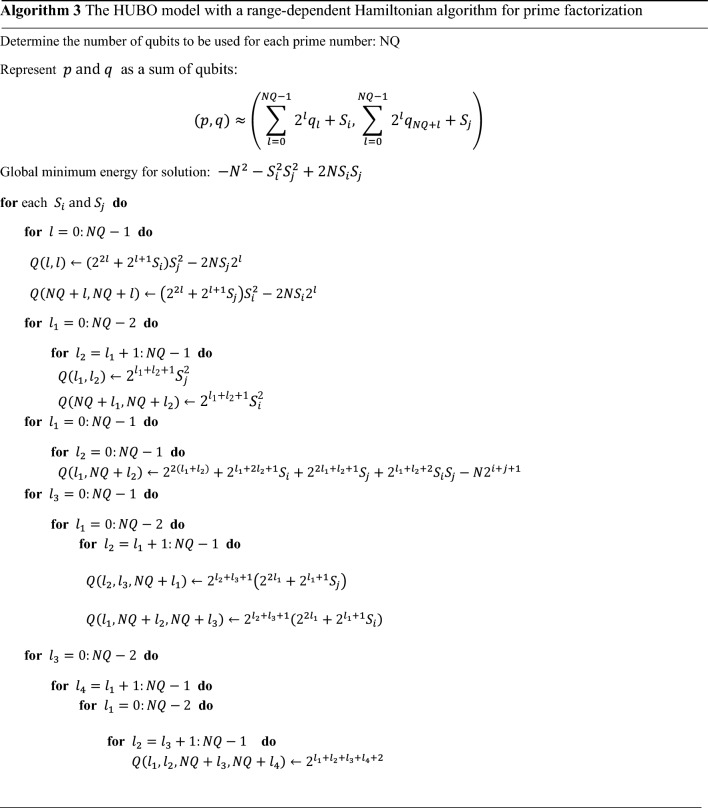


## Discussion

The proposed algorithm for prime factorization has cubic and quartic terms, so it is suitable for the HUBO model. In this paper, we factored 102,454,763 into two prime numbers, but factoring larger numbers would be possible in the future. The reason we used 26 logical qubits in the prime factorization problem is to prevent the capacity from becoming too large when receiving results from the D-Wave system. It is possible to convert a HUBO model to a QUBO model using Algorithm 2, but this requires a large number of additional qubits. We tested an example of $N=15$ for the QUBO model. We used $(p,q) = (1+2{q}_{1}+4{q}_{2},1+2{q}_{3}+4{q}_{4})$ to solve this problem. Among the additional 11 logical qubits used, 4 qubits were used when converting cubic terms into the form of the QUBO model. The remaining 7 qubits were used in the process of converting the quartic term into the QUBO model. In Fig. 1, we found that different energies appeared for the same solution $(p,q)$. This is because additional qubits were used in the process of calculating the HUBO model as a QUBO model. This does not matter because we can find the solution when the global minimum energy appears. Seven additional logical qubits are required to transform each quartic term into the terms of a QUBO model. If each $p$ and $q$ uses $n$ logical qubits, our QUBO model requires $\frac{7{n}^{2}{\left(n-1\right)}^{2}}{4}$ additional qubits for quartic terms. Therefore, our QUBO model in the current quantum annealer system is not suitable for the prime factorization of large natural numbers. If ${q}_{i}{q}_{j}{q}_{l}{q}_{m}$ can be implemented in a quantum annealer system as the concept of a delta measure ${\updelta }_{ijlm}$, where ${\updelta }_{ijlm}$ is 1 when $i=j=l=m=1$ and ${\updelta }_{ijlm}$ is zero otherwise, it is expected that there will be tremendous progress in the development of the QUBO model.

We applied the range-dependent Hamiltonian algorithm to the HUBO model. This algorithm works for any number but works better for larger numbers. The reason is that it becomes more difficult for hybrid/QPU solvers to find the global minimum energy as the number of qubits increases. By limiting the number of qubits, this algorithm can compute QUBO/HUBO models more efficiently for large numbers with a smaller number of qubits. Algorithm 3 divides the domain into several smaller intervals. The advantage of this algorithm is that it can find a solution if it can be expressed in qubits only for small intervals of change. In addition, since it can be calculated in each independent subrange, similar to the domain division of parallel computing, the more the hardware develops, the better it can be applied. We apply this algorithm to factorize 1,000,070,001,221 into 1,000,033 and 1,000,037. We used 12 logical qubits when factoring this value, and each ${S}_{i}$ and ${S}_{j}$ used 1,000,000. In general, ${S}_{i}$ and ${S}_{j}$ can be expressed as $k{2}^{n}$, but we used ${10}^{n}$ to make the calculations easier. The number 1 trillion is close to the largest number that can be factored through the D-Wave system. When calculating a number greater than 5% of the number we calculated, an error message was sent because the coefficients of the terms of the HUBO model were not allocated normally in the D-Wave system. We used the Decimal function to solve this problem, but it was not properly allocated in the D-Wave system. As another approach to a large number $N$, we calculated the double type for each coefficient but could not find the global minimum energy in the D-Wave system due to floating-point error mitigation. For example, when calculating the coefficient of a term ${q}_{{l}_{1}}{q}_{{l}_{2}}{q}_{n+{l}_{3}}{q}_{n+{l}_{4}}$ in Eq. (10), if the number of qubits is 13, then each $l$ is 12. Then, the coefficient for this term is ${2}^{50}=\mathrm{1,125,899,906,842,624}$, and for this large value, the hybrid solver cannot be implemented in the quantum annealer and displays an error message “UFuncTypeError”. It seems that there is a physical limit on the coefficients of the HUBO/QUBO model in the quantum annealer. If the number of qubits in the hybrid solver increases and the coefficients of the HUBO/QUBO model can be accurately expressed in quantum annealers, we think that the prime factorization problem will be solved.

The CPU time needed to prime factorize 102,454,763 with MATLAB on a classical computer was 0.000748 s, and the time for 1,000,070,001,221 was 0.014032 s. It is expected that it will take a few seconds to factor numbers close to ${10}^{18}$ into two prime numbers. The hybrid solver can be set for at least 3 s for one calculation. It takes approximately 60 qubits to prime factor ${10}^{18}$. In general, hybrid solvers find the minimum value well even when using hundreds of qubits and find energies similar to the minimum energy even when using 10,000 qubits^17^. Our HUBO model is predicted to outperform classical computers when the approximation coefficient ${2}^{120}$ in Eq. (10) is physically implemented in a quantum annealer (Supplementary information S1).

## Supplementary Information

Supplementary Information.

## Data Availability

All data generated or analyzed during this study are included in this published article.
