# Supplementary material for: HUBO and QUBO models for prime factorization
Source: Sci Rep. 2023 Jun 21;13:10080. doi: 10.1038/s41598-023-36813-x (PMC10284802; doi:10.1038/s41598-023-36813-x)
Supplement: Supplementary file 1 — Supplementary Information. [file 41598_2023_36813_MOESM1_ESM.zip › PF_QUBO.pdf]

In [1]:
```
##################################################
###       Prime factorization for RSA keys       ###
### Code developer: Hyunju Lee and Kyungtaek Jun ###
###     Code version 1.0 - integer variable      ###
##################################################

##### Initial setting #####
# x1 = q0 + 2q1 + 4q2 + --- + 2^(n-1)q(n-1)
# x2 = qn + 2q(n+1) + 4q(n+2) + ---  2^(n-1)q(2n-1)
# x1x2 = c

### Least square problem
# QUBO = (x1x2 - c)^2 - c^2

import numpy as np
import random, math
import copy
from dwave.system import DWaveSampler, EmbeddingComposite
sampler_auto = EmbeddingComposite(DWaveSampler(solver={'qpu': True}))

x1 = int(3)
x2 = int(5)
c = x1*x2
print ("first prime number: ",x1)
print ("second prime number: ",x2)
print ("RSA number: ",c)

qubits = 2
max_d = format(len(str(2*qubits)), '02')
size_QM = int(2*qubits+ qubits*qubits*(qubits-1) + 7*qubits*(qubits-1)*qubits*(qubits-1)/4)
QM = np.zeros((size_QM, size_QM))
ME = -c*c+2*c-1

def Q_mat(qubits,QM):
    max_d = format(len(str(qubits)), '02')

    # linear terms
    Q = {}
    for i in range(qubits):
        if QM[i][i] != 0:
            linear_term = format(i + 1, max_d)
            exec("Q.update({('q%s','q%s'):%s})"%(linear_term, linear_term, format(QM[i][i])))
```

```
    # quadratic terms
    for i in range(qubits-1):
        for j in range(i+1,qubits):
#            if QM[i][j] != 0:
            qdrt1 = format(i + 1, max_d)
            qdrt2 = format(j + 1, max_d)
            exec("Q.update({('q%s','q%s'):%s})"%(qdrt1,qdrt2,format(QM[i][j])))
    return Q
```

```
first prime number:  3
second prime number:  5
RSA number:  15
```

In [2]:

```
QM[0][0] = -52
QM[1][1] = -96
QM[2][2] = -52
QM[3][3] = -96
QM[0][1] = 16
QM[0][2] = -56
QM[0][3] = -48
QM[1][2] = -48
QM[1][3] = 96
QM[2][3] = 16

po1 = 0
po2 = 1
po3 = 2
val = 128
# existed qubits
QM[po1][po2] = QM[po1][po2] + val
QM[po1][po3] = QM[po1][po3] + val
QM[po2][po3] = QM[po2][po3] + val
QM[po1][po1] = QM[po1][po1] - val
QM[po2][po2] = QM[po2][po2] - val
QM[po3][po3] = QM[po3][po3] - val
# new qubit
po_qb = 4
QM[po1][po_qb] = QM[po1][po_qb] + val
QM[po2][po_qb] = QM[po2][po_qb] + val
QM[po3][po_qb] = QM[po3][po_qb] + val
QM[po_qb][po_qb] = QM[po_qb][po_qb] - val
ME = ME - val
```

```
po1 = 0
po2 = 1
po3 = 3
val = 384
# existed qubits
QM[po1][po2] = QM[po1][po2] + val
QM[po1][po3] = QM[po1][po3] + val
QM[po2][po3] = QM[po2][po3] + val
QM[po1][po1] = QM[po1][po1] - val
QM[po2][po2] = QM[po2][po2] - val
QM[po3][po3] = QM[po3][po3] - val
# new qubit
po_qb = 5
QM[po1][po_qb] = QM[po1][po_qb] + val
QM[po2][po_qb] = QM[po2][po_qb] + val
QM[po3][po_qb] = QM[po3][po_qb] + val
QM[po_qb][po_qb] = QM[po_qb][po_qb] - val
ME = ME - val

po1 = 0
po2 = 2
po3 = 3
val = 128
# existed qubits
QM[po1][po2] = QM[po1][po2] + val
QM[po1][po3] = QM[po1][po3] + val
QM[po2][po3] = QM[po2][po3] + val
QM[po1][po1] = QM[po1][po1] - val
QM[po2][po2] = QM[po2][po2] - val
QM[po3][po3] = QM[po3][po3] - val
# new qubit
po_qb = 6
QM[po1][po_qb] = QM[po1][po_qb] + val
QM[po2][po_qb] = QM[po2][po_qb] + val
QM[po3][po_qb] = QM[po3][po_qb] + val
QM[po_qb][po_qb] = QM[po_qb][po_qb] - val
ME = ME - val


po1 = 1
po2 = 2
po3 = 3
val = 384
# existed qubits
```

```
QM[po1][po2] = QM[po1][po2] + val
QM[po1][po3] = QM[po1][po3] + val
QM[po2][po3] = QM[po2][po3] + val
QM[po1][po1] = QM[po1][po1] - val
QM[po2][po2] = QM[po2][po2] - val
QM[po3][po3] = QM[po3][po3] - val
# new qubit
po_qb = 7
QM[po1][po_qb] = QM[po1][po_qb] + val
QM[po2][po_qb] = QM[po2][po_qb] + val
QM[po3][po_qb] = QM[po3][po_qb] + val
QM[po_qb][po_qb] = QM[po_qb][po_qb] - val
ME = ME - val

po1 = 0
po2 = 1
po3 = 2
po4 = 3
val = 256
# linear terms with existed qubits
QM[po1][po1] = QM[po1][po1] - 5*val
QM[po2][po2] = QM[po2][po2] - 3*val
QM[po3][po3] = QM[po3][po3] - 3*val
QM[po4][po4] = QM[po4][po4] - 3*val
# quadratic with existed qubits
QM[po1][po2] = QM[po1][po2] + 2*val
QM[po1][po3] = QM[po1][po3] + 2*val
QM[po1][po4] = QM[po1][po4] + 2*val
QM[po2][po3] = QM[po2][po3] + val
QM[po2][po4] = QM[po2][po4] + val
QM[po3][po4] = QM[po3][po4] + val

# linear terms with new qubits
po_qb = 8
QM[po_qb][po_qb] = QM[po_qb][po_qb] - 3*val
QM[po_qb+1][po_qb+1] = QM[po_qb+1][po_qb+1] - val
QM[po_qb+2][po_qb+2] = QM[po_qb+2][po_qb+2] - val
QM[po_qb+3][po_qb+3] = QM[po_qb+3][po_qb+3] - val
QM[po_qb+4][po_qb+4] = QM[po_qb+4][po_qb+4] - val
QM[po_qb+5][po_qb+5] = QM[po_qb+5][po_qb+5] - val
QM[po_qb+6][po_qb+6] = QM[po_qb+6][po_qb+6] - val
# quadratic with combined qubits
QM[po1][po_qb] = QM[po1][po_qb] + 2*val
QM[po1][po_qb+1] = QM[po1][po_qb+1] + val
```

```
QM[po1][po_qb+2] = QM[po1][po_qb+2] + val
QM[po1][po_qb+3] = QM[po1][po_qb+3] + val
QM[po1][po_qb+4] = QM[po1][po_qb+4] + val
QM[po1][po_qb+5] = QM[po1][po_qb+5] + val
QM[po1][po_qb+6] = QM[po1][po_qb+6] + val
QM[po2][po_qb] = QM[po2][po_qb] + val
QM[po2][po_qb+1] = QM[po2][po_qb+1] + val
QM[po2][po_qb+4] = QM[po2][po_qb+4] + val
QM[po2][po_qb+5] = QM[po2][po_qb+5] + val
QM[po3][po_qb] = QM[po3][po_qb] + val
QM[po3][po_qb+2] = QM[po3][po_qb+2] + val
QM[po3][po_qb+4] = QM[po3][po_qb+4] + val
QM[po3][po_qb+6] = QM[po3][po_qb+6] + val
QM[po4][po_qb] = QM[po4][po_qb] + val
QM[po4][po_qb+3] = QM[po4][po_qb+3] + val
QM[po4][po_qb+5] = QM[po4][po_qb+5] + val
QM[po4][po_qb+6] = QM[po4][po_qb+6] + val
# quadratic with only new qubits
QM[po_qb][po_qb+1] = QM[po_qb][po_qb+1] + val
QM[po_qb][po_qb+2] = QM[po_qb][po_qb+2] + val
QM[po_qb][po_qb+3] = QM[po_qb][po_qb+3] + val
ME = ME - 6*val
```

In [3]:

```
# Print Matrix Q
# To reduce result, you can put "#" in front of "print(QM)" and "print(sampleset)"
print("₩n")
print("# Matrix Q is")
print(QM)
print("₩nMinimum energy is ",ME)
print("₩n")

# Annealing on D-Wave system
Q = Q_mat(size_QM,QM)
sampleset = sampler_auto.sample_qubo(Q, num_reads=2000)

print("₩n")
print(sampleset.first)
print("₩n")
print(sampleset)
```

```
# Matrix Q is
[[-1972.  1040.   712.   976.   128.   384.   128.     0.   512.   256.
    256.   256.   256.   256.   256.]
 [    0. -1760.   720.  1120.   128.   384.     0.   384.   256.   256.
      0.     0.   256.   256.     0.]
 [    0.     0. -1460.   784.   128.     0.   128.   384.   256.     0.
    256.     0.   256.     0.   256.]
 [    0.     0.     0. -1760.     0.   384.   128.   384.   256.     0.
      0.   256.     0.   256.   256.]
 [    0.     0.     0.     0.  -128.     0.     0.     0.     0.     0.
      0.     0.     0.     0.     0.]
 [    0.     0.     0.     0.     0.  -384.     0.     0.     0.     0.
      0.     0.     0.     0.     0.]
 [    0.     0.     0.     0.     0.     0.  -128.     0.     0.     0.
      0.     0.     0.     0.     0.]
 [    0.     0.     0.     0.     0.     0.     0.  -384.     0.     0.
      0.     0.     0.     0.     0.]
 [    0.     0.     0.     0.     0.     0.     0.     0.  -768.   256.
    256.   256.     0.     0.     0.]
 [    0.     0.     0.     0.     0.     0.     0.     0.     0.  -256.
      0.     0.     0.     0.     0.]
 [    0.     0.     0.     0.     0.     0.     0.     0.     0.     0.
   -256.     0.     0.     0.     0.]
 [    0.     0.     0.     0.     0.     0.     0.     0.     0.     0.
      0.  -256.     0.     0.     0.]
 [    0.     0.     0.     0.     0.     0.     0.     0.     0.     0.
      0.     0.  -256.     0.     0.]
 [    0.     0.     0.     0.     0.     0.     0.     0.     0.     0.
      0.     0.     0.  -256.     0.]
 [    0.     0.     0.     0.     0.     0.     0.     0.     0.     0.
      0.     0.     0.     0.  -256.]]

Minimum energy is  -2756
```

```
Sample(sample={'q01': 0, 'q02': 1, 'q03': 1, 'q04': 0, 'q05': 0, 'q06': 0, 'q07': 0, 'q08': 0, 'q09': 1, 'q10': 0, 'q11': 0, 'q12': 0, 'q13': 0, 'q14': 0, 'q15': 0}, energy=-2756.0, num_occurrences=33, chain_break_fraction=0.06666666666666667)
```

```
    q01 q02 q03 q04 q05 q06 q07 q08 q09 q10 q11 q12 ... q15  energy num_oc. ...
0     0   1   1   0   0   0   0   0   1   0   0   0 ...   0 -2756.0      33 ...
```

1	0	1	1	0	0	0	0	0	1	0	0	0	...	1	-2756.0	75	...
18	0	1	1	0	0	0	1	0	1	0	0	0	...	0	-2756.0	30	...
19	0	1	1	0	0	0	1	0	1	0	0	0	...	1	-2756.0	47	...
20	0	1	1	0	0	0	1	0	1	0	0	0	...	0	-2756.0	14	...
21	0	1	1	0	0	1	1	0	1	0	0	0	...	0	-2756.0	4	...
22	0	1	1	0	0	1	0	0	1	0	0	0	...	0	-2756.0	8	...
23	0	1	1	0	0	0	0	0	1	0	0	0	...	0	-2756.0	2	...
24	0	1	1	0	0	0	0	0	1	0	0	0	...	1	-2756.0	5	...
25	0	1	1	0	0	0	1	0	1	0	0	0	...	1	-2756.0	27	...
26	0	1	1	0	0	1	0	0	1	0	0	0	...	1	-2756.0	14	...
27	0	1	1	0	0	1	1	0	1	0	0	0	...	1	-2756.0	18	...
36	0	1	1	0	0	0	0	0	1	0	0	0	...	1	-2756.0	4	...
37	0	1	1	0	0	0	0	0	1	0	0	0	...	0	-2756.0	1	...
83	0	1	1	0	0	0	1	0	1	0	0	0	...	1	-2756.0	1	...
84	0	1	1	0	0	0	1	0	1	0	0	0	...	0	-2756.0	1	...
96	1	0	0	1	0	0	0	1	0	1	1	0	...	0	-2756.0	2	...
97	0	1	1	0	0	0	0	0	1	0	0	1	...	0	-2756.0	7	...
98	0	1	1	0	0	1	1	0	1	0	0	0	...	0	-2756.0	1	...
99	1	0	0	1	0	0	0	1	0	1	0	0	...	0	-2756.0	1	...
100	0	1	1	0	0	1	0	0	1	0	0	1	...	0	-2756.0	1	...
101	1	0	0	1	0	0	0	0	1	0	0	0	...	0	-2756.0	1	...
102	1	0	0	1	0	0	0	1	0	1	0	0	...	0	-2756.0	1	...
103	0	1	1	0	0	0	0	0	1	0	0	0	...	0	-2756.0	2	...
104	1	0	0	1	1	0	0	1	0	0	1	0	...	0	-2756.0	2	...
105	1	0	0	1	1	0	0	1	1	0	0	0	...	0	-2756.0	1	...
106	0	1	1	0	0	0	1	0	0	1	1	1	...	0	-2756.0	1	...
107	0	1	1	0	0	0	1	0	0	1	1	1	...	0	-2756.0	1	...
108	0	1	1	0	0	1	0	0	1	0	0	0	...	0	-2756.0	2	...
109	1	0	0	1	1	0	0	1	0	0	0	0	...	0	-2756.0	1	...
110	1	0	0	1	0	0	0	1	0	0	0	0	...	0	-2756.0	2	...
111	0	1	1	0	0	0	0	0	0	1	0	1	...	0	-2756.0	1	...
112	0	1	1	0	0	0	0	0	0	0	1	1	...	0	-2756.0	1	...
113	0	1	1	0	0	0	1	0	1	0	0	0	...	0	-2756.0	1	...
114	0	1	1	0	0	0	0	0	0	0	0	1	...	0	-2756.0	1	...
115	0	1	1	0	0	0	1	0	0	0	1	1	...	0	-2756.0	3	...
116	0	1	1	0	0	1	1	0	0	0	0	1	...	0	-2756.0	2	...
117	0	1	1	0	0	0	1	0	1	0	0	1	...	0	-2756.0	6	...
118	0	1	1	0	0	0	1	0	1	0	0	0	...	0	-2756.0	3	...
119	1	0	0	1	1	0	0	0	0	0	0	0	...	0	-2756.0	1	...
120	0	1	1	0	0	1	0	0	1	0	0	1	...	0	-2756.0	1	...
121	0	1	1	0	0	0	1	0	0	0	0	1	...	0	-2756.0	1	...
122	0	1	1	0	0	0	0	0	1	0	0	1	...	0	-2756.0	3	...
123	0	1	1	0	0	0	1	0	0	0	0	1	...	1	-2756.0	2	...
124	0	1	1	0	0	0	1	0	1	0	0	1	...	1	-2756.0	8	...

125	0	1	1	0	0	0	1	0	0	1	1	1	...	1	-2756.0	1	...
126	0	1	1	0	0	1	0	0	0	1	0	1	...	1	-2756.0	1	...
127	0	1	1	0	0	1	0	0	0	0	0	1	...	1	-2756.0	2	...
128	0	1	1	0	0	0	0	0	1	0	0	1	...	1	-2756.0	7	...
129	0	1	1	0	0	1	1	0	0	0	1	1	...	0	-2756.0	1	...
130	0	1	1	0	0	1	1	0	0	0	0	1	...	1	-2756.0	2	...
131	0	1	1	0	0	0	1	0	1	0	0	0	...	1	-2756.0	3	...
132	0	1	1	0	0	1	1	0	1	0	0	1	...	1	-2756.0	3	...
133	0	1	1	0	0	1	1	0	0	1	1	1	...	1	-2756.0	1	...
134	0	1	1	0	0	1	0	0	0	1	1	1	...	1	-2756.0	1	...
135	0	1	1	0	0	0	0	0	0	1	0	1	...	1	-2756.0	1	...
136	0	1	1	0	0	1	1	0	0	0	1	1	...	1	-2756.0	1	...
137	0	1	1	0	0	0	1	0	0	0	1	1	...	1	-2756.0	4	...
138	0	1	1	0	0	0	1	0	1	0	0	0	...	1	-2756.0	9	...
139	0	1	1	0	0	1	1	0	1	0	0	0	...	1	-2756.0	6	...
140	0	1	1	0	0	1	0	0	0	1	1	1	...	1	-2756.0	1	...
141	0	1	1	0	0	0	1	0	1	0	0	1	...	0	-2756.0	3	...
142	0	1	1	0	0	0	0	0	0	0	1	1	...	1	-2756.0	2	...
143	0	1	1	0	0	0	0	0	1	0	0	0	...	1	-2756.0	4	...
144	0	1	1	0	0	0	0	0	0	1	1	1	...	1	-2756.0	1	...
145	0	1	1	0	0	0	0	0	0	0	0	1	...	1	-2756.0	2	...
146	0	1	1	0	0	0	0	0	0	1	0	1	...	1	-2756.0	1	...
147	0	1	1	0	0	0	0	0	0	0	0	1	...	1	-2756.0	1	...
148	0	1	1	0	0	1	1	0	0	0	0	1	...	0	-2756.0	1	...
149	0	1	1	0	0	0	0	0	0	0	1	1	...	1	-2756.0	1	...
150	0	1	1	0	0	0	0	0	1	0	0	1	...	1	-2756.0	12	...
151	0	1	1	0	0	1	1	0	1	0	0	1	...	0	-2756.0	4	...
152	0	1	1	0	0	0	1	0	0	0	0	1	...	0	-2756.0	1	...
153	0	1	1	0	0	1	1	0	0	1	1	1	...	1	-2756.0	1	...
154	0	1	1	0	0	1	0	0	1	0	0	0	...	1	-2756.0	3	...
155	0	1	1	0	0	1	1	0	1	0	0	1	...	1	-2756.0	6	...
156	0	1	1	0	0	0	1	0	0	1	0	1	...	1	-2756.0	1	...
157	0	1	1	0	0	1	1	0	0	0	0	1	...	1	-2756.0	1	...
158	0	1	1	0	0	0	1	0	0	0	0	1	...	1	-2756.0	3	...
159	0	1	1	0	0	0	1	0	1	0	0	1	...	1	-2756.0	8	...
174	0	1	1	0	0	0	1	0	1	0	0	0	...	1	-2756.0	1	...
175	0	1	1	0	0	1	1	0	1	0	0	0	...	1	-2756.0	1	...
361	0	1	1	0	0	0	0	0	1	0	0	1	...	1	-2756.0	1	...
362	0	1	1	0	0	0	0	0	1	0	0	0	...	1	-2756.0	1	...
365	0	1	1	0	0	0	0	0	0	0	0	1	...	1	-2756.0	1	...
415	0	1	1	0	0	0	0	0	1	0	0	0	...	1	-2756.0	1	...
537	0	1	1	0	0	0	1	0	1	0	0	1	...	0	-2756.0	1	...
656	0	1	1	0	0	0	1	0	1	0	0	1	...	1	-2756.0	1	...
657	0	1	1	0	0	0	1	0	0	1	0	1	...	1	-2756.0	1	...

763	0	1	1	0	0	0	0	0	1	0	0	0	...	1	-2756.0	1	...
864	0	1	1	0	0	0	1	0	1	0	0	0	...	0	-2756.0	1	...
866	0	1	1	0	0	0	1	0	1	0	0	1	...	0	-2756.0	1	...
867	0	1	1	0	0	1	1	0	1	0	0	0	...	1	-2756.0	1	...
878	0	1	1	0	0	1	1	0	1	0	0	0	...	0	-2756.0	1	...
880	0	1	1	0	0	0	0	0	1	0	0	1	...	1	-2756.0	1	...
883	0	1	1	0	0	0	0	0	1	0	0	1	...	1	-2756.0	1	...
312	1	0	1	0	0	0	0	0	0	0	0	0	...	0	-2720.0	1	...
313	1	0	1	0	0	1	0	0	0	0	0	1	...	0	-2720.0	1	...
314	1	0	1	0	0	0	0	0	0	0	0	1	...	0	-2720.0	1	...
315	1	0	1	0	0	1	0	1	1	0	0	0	...	0	-2720.0	1	...
316	1	0	1	0	0	0	0	1	0	1	0	1	...	0	-2720.0	1	...
317	1	0	1	0	0	0	0	0	0	0	0	0	...	0	-2720.0	1	...
318	1	0	1	0	0	0	0	1	0	0	0	1	...	0	-2720.0	1	...
57	0	0	1	1	0	0	0	0	0	1	0	0	...	0	-2692.0	1	...
58	0	0	1	1	0	0	0	0	1	0	0	0	...	0	-2692.0	9	...
59	0	0	1	1	0	0	0	0	0	1	1	0	...	0	-2692.0	1	...
60	0	0	1	1	0	0	0	0	1	1	0	0	...	0	-2692.0	7	...
61	0	0	1	1	0	0	0	0	0	1	1	1	...	0	-2692.0	2	...
62	0	0	1	1	0	0	0	0	1	0	0	0	...	0	-2692.0	21	...
63	0	0	1	1	1	0	0	0	0	1	0	0	...	0	-2692.0	2	...
64	0	0	1	1	0	0	0	0	0	1	0	0	...	0	-2692.0	2	...
65	0	0	1	1	1	0	0	0	0	1	0	1	...	0	-2692.0	3	...
66	0	0	1	1	1	0	0	0	0	1	1	0	...	0	-2692.0	1	...
67	0	0	1	1	1	0	0	0	1	1	0	0	...	0	-2692.0	5	...
68	0	0	1	1	0	0	0	0	0	1	0	1	...	0	-2692.0	4	...
69	0	0	1	1	1	0	0	0	1	0	0	0	...	0	-2692.0	9	...
70	0	0	1	1	1	0	0	0	0	1	0	1	...	0	-2692.0	3	...
71	0	0	1	1	0	0	0	0	1	1	0	0	...	0	-2692.0	15	...
72	0	0	1	1	1	0	0	0	0	1	1	1	...	0	-2692.0	1	...
73	0	0	1	1	1	0	0	0	1	0	0	0	...	0	-2692.0	13	...
74	0	0	1	1	0	0	0	0	0	1	0	1	...	0	-2692.0	1	...
75	0	0	1	1	1	0	0	0	0	1	1	1	...	0	-2692.0	3	...
76	0	0	1	1	1	0	0	0	1	1	0	0	...	0	-2692.0	10	...
77	0	0	1	1	1	0	0	0	0	1	0	0	...	0	-2692.0	1	...
176	0	0	1	1	0	0	0	0	1	0	0	0	...	0	-2692.0	1	...
177	0	0	1	1	0	0	0	0	1	0	0	0	...	0	-2692.0	5	...
178	0	0	1	1	0	0	0	0	1	0	0	0	...	0	-2692.0	3	...
179	0	0	1	1	0	0	0	0	1	0	0	0	...	0	-2692.0	1	...
180	0	0	1	1	0	0	0	0	1	1	0	0	...	0	-2692.0	2	...
181	0	0	1	1	0	0	0	0	1	1	0	0	...	0	-2692.0	2	...
182	0	0	1	1	0	0	0	0	1	1	0	0	...	0	-2692.0	1	...
183	0	0	1	1	0	0	0	0	0	1	0	1	...	0	-2692.0	1	...
184	0	0	1	1	0	0	0	0	0	1	0	1	...	0	-2692.0	1	...

329	0	0	1	1	0	0	0	0	1	0	0	0	...	0	-2692.0	1	...
330	0	0	1	1	0	0	0	0	1	0	0	0	...	0	-2692.0	1	...
331	0	0	1	1	0	0	0	0	1	1	0	0	...	0	-2692.0	1	...
332	0	0	1	1	1	1	0	0	1	0	0	0	...	0	-2692.0	3	...
333	0	0	1	1	1	1	0	0	1	1	0	0	...	0	-2692.0	1	...
334	0	0	1	1	0	1	0	0	0	1	0	1	...	0	-2692.0	2	...
335	0	0	1	1	1	0	0	0	1	1	0	0	...	0	-2692.0	1	...
336	0	0	1	1	1	1	0	0	1	0	0	0	...	0	-2692.0	1	...
337	0	0	1	1	0	1	0	0	1	0	0	0	...	0	-2692.0	1	...
338	0	0	1	1	0	1	0	0	1	1	0	0	...	0	-2692.0	1	...
339	0	0	1	1	1	0	0	0	1	0	0	0	...	0	-2692.0	1	...
340	0	0	1	1	1	0	0	0	1	1	0	0	...	0	-2692.0	2	...
341	0	0	1	1	1	0	0	0	0	1	0	1	...	0	-2692.0	1	...
342	0	0	1	1	1	1	0	0	1	1	0	0	...	0	-2692.0	2	...
343	0	0	1	1	1	1	0	0	0	1	1	1	...	0	-2692.0	1	...
344	0	0	1	1	0	0	0	0	1	0	0	0	...	0	-2692.0	1	...
348	0	0	1	1	1	0	0	0	1	0	0	0	...	0	-2692.0	4	...
349	0	0	1	1	1	0	0	0	1	1	0	0	...	0	-2692.0	3	...
352	0	0	1	1	1	0	0	0	1	1	0	0	...	0	-2692.0	1	...
366	0	0	1	1	0	1	0	0	0	1	1	1	...	0	-2692.0	1	...
367	0	0	1	1	0	1	0	0	1	0	0	0	...	0	-2692.0	1	...
368	0	0	1	1	0	1	0	0	0	1	0	0	...	0	-2692.0	2	...
369	0	0	1	1	0	0	0	0	1	1	0	0	...	0	-2692.0	1	...
370	0	0	1	1	0	1	0	0	1	1	0	0	...	0	-2692.0	1	...
371	0	0	1	1	0	1	0	0	1	0	0	0	...	0	-2692.0	1	...
372	0	0	1	1	0	0	0	0	0	1	1	0	...	0	-2692.0	1	...
373	0	0	1	1	0	0	0	0	0	1	0	1	...	0	-2692.0	1	...
374	0	0	1	1	1	0	0	0	0	1	1	0	...	0	-2692.0	1	...
375	0	0	1	1	0	1	0	0	0	1	1	1	...	0	-2692.0	1	...
376	0	0	1	1	0	1	0	0	1	0	0	0	...	0	-2692.0	2	...
377	0	0	1	1	0	0	0	0	1	1	0	0	...	0	-2692.0	1	...
378	0	0	1	1	0	0	0	0	1	1	0	0	...	0	-2692.0	1	...
379	0	0	1	1	0	0	0	0	1	0	0	0	...	0	-2692.0	1	...
380	0	0	1	1	0	0	0	0	1	1	0	0	...	0	-2692.0	1	...
381	1	1	0	0	0	0	1	1	0	0	1	1	...	0	-2692.0	3	...
382	0	0	1	1	1	0	0	0	1	0	0	0	...	0	-2692.0	5	...
383	0	0	1	1	1	0	0	0	1	1	0	0	...	0	-2692.0	1	...
384	1	1	0	0	0	0	0	0	0	0	1	1	...	0	-2692.0	3	...
385	0	0	1	1	1	1	0	0	1	0	0	0	...	0	-2692.0	1	...
386	0	0	1	1	1	1	0	0	0	1	0	0	...	0	-2692.0	2	...
387	0	0	1	1	1	1	0	0	0	1	0	1	...	0	-2692.0	1	...
388	1	1	0	0	0	0	1	1	1	0	0	0	...	0	-2692.0	2	...
389	0	0	1	1	1	0	0	0	0	1	0	1	...	0	-2692.0	3	...
390	0	0	1	1	1	0	0	0	1	0	0	0	...	0	-2692.0	2	...

391	1	1	0	0	0	0	1	1	0	0	0	0	...	0	-2692.0	1	...
392	0	0	1	1	1	0	0	0	1	0	0	0	...	0	-2692.0	1	...
393	1	1	0	0	0	0	1	1	0	0	1	0	...	0	-2692.0	4	...
394	1	1	0	0	0	0	0	0	0	0	1	0	...	0	-2692.0	2	...
395	1	1	0	0	0	0	1	0	0	0	1	0	...	0	-2692.0	1	...
396	0	0	1	1	1	0	0	0	1	1	0	0	...	0	-2692.0	2	...
397	0	0	1	1	1	1	0	0	1	1	0	0	...	0	-2692.0	1	...
398	0	0	1	1	1	0	0	0	1	0	0	0	...	0	-2692.0	1	...
399	1	1	0	0	0	0	0	1	0	0	1	1	...	0	-2692.0	4	...
400	1	1	0	0	0	0	0	1	0	0	1	0	...	0	-2692.0	3	...
401	1	1	0	0	0	0	1	1	0	0	1	1	...	1	-2692.0	3	...
402	1	1	0	0	0	0	0	1	0	0	0	1	...	0	-2692.0	5	...
403	1	1	0	0	0	0	1	1	0	0	0	1	...	0	-2692.0	3	...
404	1	1	0	0	0	0	1	0	0	0	1	1	...	0	-2692.0	5	...
405	1	1	0	0	0	0	0	1	0	0	1	1	...	1	-2692.0	2	...
406	1	1	0	0	0	0	0	1	0	0	1	0	...	1	-2692.0	2	...
407	1	1	0	0	0	0	1	1	0	0	1	0	...	1	-2692.0	1	...
408	1	1	0	0	0	0	0	1	0	0	0	0	...	0	-2692.0	2	...
409	1	1	0	0	0	0	0	0	0	0	0	1	...	0	-2692.0	1	...
410	1	1	0	0	0	0	0	0	0	0	1	1	...	1	-2692.0	1	...
411	1	1	0	0	0	0	1	0	0	0	0	1	...	0	-2692.0	1	...
412	1	1	0	0	0	0	0	1	0	0	0	1	...	1	-2692.0	1	...
413	1	1	0	0	0	0	1	0	0	0	1	1	...	1	-2692.0	1	...
414	1	1	0	0	0	0	1	1	0	0	0	1	...	1	-2692.0	3	...
435	1	1	0	0	0	0	0	1	0	0	1	0	...	0	-2692.0	1	...
438	1	1	0	0	0	0	1	1	0	0	1	0	...	0	-2692.0	2	...
445	1	1	0	0	0	0	0	1	0	0	1	1	...	0	-2692.0	2	...
448	1	1	0	0	0	0	0	1	0	0	0	1	...	0	-2692.0	2	...
450	1	1	0	0	0	0	1	1	1	0	0	0	...	0	-2692.0	1	...
454	1	1	0	0	0	0	1	1	0	0	1	1	...	0	-2692.0	6	...
458	1	1	0	0	0	0	1	1	0	0	1	1	...	1	-2692.0	1	...
460	1	1	0	0	0	0	1	1	0	0	0	1	...	0	-2692.0	2	...
461	1	1	0	0	0	0	0	1	1	0	0	0	...	1	-2692.0	1	...
462	1	1	0	0	0	0	1	1	0	0	0	1	...	1	-2692.0	1	...
466	1	1	0	0	0	0	1	1	0	0	1	1	...	0	-2692.0	1	...
484	1	1	0	0	0	0	1	1	0	0	0	1	...	0	-2692.0	1	...
485	1	1	0	0	0	0	0	1	0	0	0	0	...	0	-2692.0	1	...
513	0	0	1	1	0	0	0	0	1	1	0	0	...	0	-2692.0	1	...
514	0	0	1	1	0	0	0	0	1	1	0	0	...	0	-2692.0	1	...
515	0	0	1	1	0	0	0	0	1	0	0	0	...	0	-2692.0	2	...
568	0	0	1	1	1	0	0	0	1	0	0	0	...	0	-2692.0	1	...
570	0	0	1	1	0	1	0	0	1	1	0	0	...	0	-2692.0	1	...
572	0	0	1	1	1	0	0	0	1	1	0	0	...	0	-2692.0	1	...
613	0	0	1	1	0	0	0	0	1	1	0	0	...	0	-2692.0	1	...

628	0	0	1	1	1	0	0	0	1	0	0	0	...	0	-2692.0	1	...
633	0	0	1	1	1	1	0	0	1	0	0	0	...	0	-2692.0	1	...
746	1	1	0	0	0	0	0	1	0	0	0	0	...	0	-2692.0	1	...
871	0	0	1	1	0	0	0	0	1	0	0	0	...	0	-2692.0	1	...
78	0	1	0	0	0	0	1	0	1	0	1	0	...	1	-2656.0	1	...
79	0	1	0	0	0	0	1	0	1	0	0	0	...	1	-2656.0	4	...
80	0	1	0	0	0	0	1	0	1	0	1	0	...	1	-2656.0	1	...
81	0	1	0	0	1	0	1	0	1	0	0	0	...	1	-2656.0	1	...
82	0	1	0	0	1	0	1	0	1	0	1	0	...	1	-2656.0	1	...
85	0	0	0	1	1	0	1	0	1	0	1	0	...	0	-2656.0	2	...
86	0	0	0	1	1	0	0	0	1	0	1	0	...	0	-2656.0	1	...
87	0	0	0	1	1	0	0	0	0	1	1	1	...	1	-2656.0	1	...
88	0	0	0	1	1	0	1	0	1	0	0	0	...	1	-2656.0	1	...
89	0	0	0	1	1	0	0	0	1	0	1	0	...	1	-2656.0	1	...
90	0	0	0	1	1	0	1	0	0	1	1	0	...	1	-2656.0	1	...
91	0	0	0	1	1	0	1	0	1	1	1	0	...	1	-2656.0	1	...
92	0	0	0	1	1	0	1	0	1	0	1	0	...	1	-2656.0	1	...
93	0	0	0	1	1	0	0	0	1	0	0	0	...	1	-2656.0	2	...
160	0	1	0	0	0	1	1	0	1	0	0	0	...	1	-2656.0	1	...
161	0	1	0	0	0	1	1	0	1	0	1	0	...	1	-2656.0	1	...
162	0	1	0	0	0	1	1	0	1	0	0	0	...	1	-2656.0	1	...
163	0	1	0	0	0	0	1	0	1	0	0	0	...	1	-2656.0	1	...
164	0	1	0	0	1	1	1	0	1	0	1	0	...	1	-2656.0	1	...
165	0	1	0	0	1	0	1	0	1	0	1	0	...	1	-2656.0	1	...
429	0	0	0	1	1	1	1	0	1	0	1	0	...	1	-2656.0	1	...
430	0	0	0	1	1	0	1	0	0	1	1	1	...	0	-2656.0	1	...
493	0	1	0	1	0	0	1	0	1	0	0	0	...	0	-2656.0	1	...
494	0	0	0	1	1	0	0	0	0	1	1	1	...	1	-2656.0	1	...
495	0	0	0	1	1	0	1	0	0	1	1	0	...	0	-2656.0	1	...
496	0	1	0	0	0	0	1	1	1	0	1	1	...	1	-2656.0	1	...
497	0	1	0	0	1	0	1	0	1	0	0	0	...	1	-2656.0	1	...
498	0	1	0	0	0	0	1	1	1	0	0	0	...	1	-2656.0	1	...
499	0	1	0	0	0	0	1	1	1	0	0	0	...	1	-2656.0	1	...
500	0	1	0	0	0	0	1	1	0	0	1	1	...	1	-2656.0	1	...
501	0	1	0	1	1	0	1	0	0	1	1	1	...	0	-2656.0	1	...
502	0	1	0	0	0	0	1	1	1	0	1	1	...	1	-2656.0	1	...
503	0	1	0	0	0	1	1	0	1	0	1	1	...	1	-2656.0	1	...
504	0	0	0	1	1	0	0	1	1	0	0	0	...	1	-2656.0	1	...
505	0	0	0	1	1	1	1	0	0	1	1	1	...	1	-2656.0	1	...
506	0	0	0	1	1	0	0	1	1	1	1	0	...	1	-2656.0	1	...
507	0	1	0	0	0	1	1	0	1	0	1	0	...	1	-2656.0	1	...
508	0	1	0	0	0	0	1	1	1	0	0	0	...	1	-2656.0	1	...
509	0	0	0	1	1	1	0	0	1	0	1	0	...	1	-2656.0	1	...
510	0	1	0	0	1	0	1	0	1	0	0	1	...	1	-2656.0	1	...

511	0	1	0	0	1	0	1	1	0	0	1	1	...	1	-2656.0	1	...
512	0	1	0	0	1	0	1	0	1	0	0	1	...	1	-2656.0	1	...
874	0	0	0	1	1	0	0	1	1	0	0	0	...	1	-2656.0	1	...
38	0	1	1	0	1	0	0	0	1	0	0	0	...	0	-2628.0	7	...
39	0	1	1	0	1	0	0	0	1	0	0	0	...	1	-2628.0	6	...
94	0	1	1	0	1	0	1	0	1	0	0	0	...	0	-2628.0	5	...
95	0	1	1	0	1	0	1	0	1	0	0	0	...	1	-2628.0	9	...
303	0	1	1	0	1	1	1	0	1	0	0	0	...	0	-2628.0	1	...
304	0	1	1	0	1	0	0	0	1	0	0	0	...	0	-2628.0	1	...
305	0	1	1	0	1	0	1	0	1	0	0	0	...	0	-2628.0	1	...
306	0	1	1	0	1	1	0	0	1	0	0	0	...	0	-2628.0	2	...
307	0	1	1	0	1	1	0	0	1	0	0	0	...	1	-2628.0	1	...
308	0	1	1	0	1	1	1	0	1	0	0	0	...	1	-2628.0	4	...
309	0	1	1	0	1	0	1	0	1	0	0	0	...	1	-2628.0	3	...
351	0	1	1	0	1	0	0	0	1	0	0	0	...	0	-2628.0	1	...
353	0	1	1	0	1	0	0	0	1	0	0	0	...	1	-2628.0	1	...
547	1	0	0	1	0	0	1	1	0	1	0	0	...	0	-2628.0	1	...
548	1	0	0	1	0	0	1	0	0	1	0	0	...	0	-2628.0	1	...
549	1	0	0	1	0	0	1	1	1	0	0	0	...	0	-2628.0	1	...
550	1	0	0	1	0	0	1	1	0	0	0	0	...	0	-2628.0	1	...
551	0	1	1	0	1	1	1	0	0	1	0	1	...	0	-2628.0	1	...
552	1	0	0	1	1	0	1	1	1	0	0	0	...	0	-2628.0	1	...
553	0	1	1	0	1	0	1	0	1	0	0	1	...	0	-2628.0	1	...
554	0	1	1	0	1	0	0	0	1	0	0	0	...	1	-2628.0	1	...
555	0	1	1	0	1	0	1	0	1	0	0	0	...	0	-2628.0	3	...
556	0	1	1	0	1	0	0	0	0	1	1	1	...	1	-2628.0	1	...
557	0	1	1	0	1	0	1	0	1	0	0	1	...	1	-2628.0	2	...
558	0	1	1	0	1	1	1	0	1	0	0	1	...	1	-2628.0	3	...
559	0	1	1	0	1	0	1	0	1	0	0	1	...	1	-2628.0	2	...
560	0	1	1	0	1	0	0	0	1	0	0	1	...	1	-2628.0	1	...
561	0	1	1	0	1	1	1	0	1	0	0	0	...	1	-2628.0	1	...
562	0	1	1	0	1	0	0	0	1	0	0	1	...	1	-2628.0	1	...
563	0	1	1	0	1	1	1	0	1	0	0	1	...	1	-2628.0	1	...
564	0	1	1	0	1	0	1	0	1	0	0	0	...	1	-2628.0	1	...
565	0	1	1	0	1	0	1	0	1	0	0	0	...	1	-2628.0	2	...
749	0	1	1	0	1	0	1	0	0	0	0	1	...	1	-2628.0	1	...
3	0	0	1	0	0	1	0	0	1	0	0	0	...	1	-2612.0	1	...
6	0	0	1	0	0	1	0	0	1	0	0	0	...	0	-2612.0	5	...
7	0	0	1	0	0	1	0	0	1	0	0	1	...	0	-2612.0	3	...
10	0	0	1	0	0	1	0	1	1	0	0	0	...	0	-2612.0	1	...
13	0	0	1	0	0	1	0	1	1	0	0	1	...	0	-2612.0	4	...
15	0	0	1	0	0	1	0	1	1	0	0	1	...	1	-2612.0	3	...
16	0	0	1	0	0	1	0	1	1	0	0	0	...	1	-2612.0	3	...
17	0	0	1	0	0	1	0	0	1	0	0	1	...	1	-2612.0	5	...

28	1	0	0	0	0	0	0	1	1	0	0	0	...	0	-2612.0	1	...
29	1	0	0	0	0	0	0	1	1	0	0	0	...	0	-2612.0	2	...
30	1	0	0	0	0	1	0	1	1	0	0	0	...	0	-2612.0	3	...
31	1	0	0	0	0	1	0	1	1	0	0	0	...	0	-2612.0	3	...
32	1	0	0	0	0	0	0	1	1	0	0	0	...	1	-2612.0	2	...
33	1	0	0	0	0	0	0	1	1	0	0	0	...	1	-2612.0	1	...
34	1	0	0	0	0	1	0	1	1	0	0	0	...	1	-2612.0	3	...
35	1	0	0	0	0	1	0	1	1	0	0	0	...	1	-2612.0	4	...
40	0	0	1	0	0	1	1	1	1	0	0	0	...	0	-2612.0	3	...
44	0	0	1	0	0	1	1	0	1	0	0	0	...	0	-2612.0	2	...
45	0	0	1	0	0	1	1	0	1	0	0	1	...	0	-2612.0	1	...
47	0	0	1	0	0	1	1	1	1	0	0	1	...	1	-2612.0	1	...
48	0	0	1	0	0	1	1	0	1	0	0	0	...	1	-2612.0	2	...
51	0	0	1	0	0	1	1	1	1	0	0	0	...	1	-2612.0	2	...
52	0	0	1	0	1	1	0	0	1	0	0	0	...	1	-2612.0	1	...
53	0	0	1	0	0	1	1	0	1	0	0	1	...	1	-2612.0	1	...
54	0	0	1	0	1	1	0	0	1	0	0	1	...	0	-2612.0	1	...
55	0	0	1	0	1	1	0	1	1	0	0	1	...	1	-2612.0	1	...
166	1	0	0	0	0	0	0	1	1	0	0	0	...	0	-2612.0	1	...
167	1	0	0	0	0	0	1	1	1	0	0	0	...	0	-2612.0	2	...
168	1	0	0	0	0	0	1	1	1	0	0	0	...	0	-2612.0	2	...
169	1	0	0	0	0	1	1	1	1	0	0	0	...	0	-2612.0	1	...
170	1	0	0	0	0	1	1	1	1	0	0	0	...	0	-2612.0	6	...
171	1	0	0	0	0	1	1	1	1	0	0	0	...	1	-2612.0	2	...
172	1	0	0	0	0	1	0	1	1	0	0	0	...	1	-2612.0	1	...
173	1	0	0	0	0	1	1	1	1	0	0	0	...	1	-2612.0	1	...
201	1	0	0	0	0	0	1	1	1	0	0	0	...	0	-2612.0	1	...
203	1	0	0	0	0	0	1	1	1	0	0	0	...	0	-2612.0	1	...
204	1	0	0	0	0	1	0	1	1	0	0	0	...	0	-2612.0	1	...
218	1	0	0	0	0	1	0	1	1	0	0	0	...	0	-2612.0	3	...
220	1	0	0	0	0	1	1	1	1	0	0	0	...	0	-2612.0	2	...
227	1	0	0	0	0	0	1	1	1	0	0	0	...	1	-2612.0	1	...
233	1	0	0	0	1	1	0	1	1	0	0	0	...	0	-2612.0	1	...
234	1	0	0	0	1	1	1	1	1	0	0	0	...	0	-2612.0	2	...
244	1	0	0	0	1	0	0	1	1	0	0	0	...	0	-2612.0	1	...
245	1	0	0	0	1	0	1	1	1	0	0	0	...	0	-2612.0	1	...
253	1	0	0	0	0	1	1	1	1	0	0	0	...	0	-2612.0	3	...
258	1	0	0	0	1	1	1	1	1	0	0	0	...	0	-2612.0	1	...
264	1	0	0	0	0	1	1	1	1	0	0	0	...	1	-2612.0	1	...
266	1	0	0	0	1	0	0	1	1	0	0	0	...	1	-2612.0	1	...
284	1	0	0	0	1	1	0	1	1	0	0	0	...	1	-2612.0	1	...
286	1	0	0	0	1	0	0	1	1	0	0	0	...	1	-2612.0	3	...
319	1	0	0	0	0	0	1	1	1	0	0	0	...	0	-2612.0	1	...
320	1	0	0	0	1	1	0	1	1	0	0	0	...	0	-2612.0	1	...

321	1	0	0	0	0	0	1	1	1	0	0	0	...	1	-2612.0	1	...
322	1	0	0	0	1	0	0	1	1	0	0	0	...	0	-2612.0	1	...
323	1	0	0	0	0	1	1	1	1	0	0	0	...	0	-2612.0	4	...
324	1	0	0	0	0	1	1	1	1	0	0	0	...	1	-2612.0	3	...
325	1	0	0	0	1	1	0	1	1	0	0	0	...	1	-2612.0	1	...
326	1	0	0	0	0	1	1	1	1	0	0	0	...	1	-2612.0	2	...
327	1	0	0	0	1	0	0	1	1	0	0	0	...	1	-2612.0	1	...
328	1	0	0	0	1	1	0	1	1	0	0	0	...	1	-2612.0	2	...
416	0	0	1	0	0	1	0	0	1	0	0	0	...	0	-2612.0	1	...
418	0	0	1	0	0	1	0	0	1	0	0	1	...	0	-2612.0	1	...
421	0	0	1	0	1	1	1	0	1	0	0	0	...	1	-2612.0	1	...
424	0	0	1	0	1	1	1	1	1	0	0	0	...	0	-2612.0	1	...
425	0	0	1	0	0	1	0	0	1	1	0	1	...	1	-2612.0	1	...
426	0	0	1	0	1	1	1	1	1	0	0	1	...	0	-2612.0	1	...
427	0	0	1	0	0	1	0	0	1	0	0	1	...	1	-2612.0	2	...
428	0	0	1	0	0	1	0	1	1	0	0	1	...	1	-2612.0	2	...
566	1	0	0	0	1	1	0	1	1	0	0	0	...	1	-2612.0	1	...
579	0	0	1	0	0	1	1	0	1	1	0	0	...	0	-2612.0	1	...
580	0	0	1	0	0	1	0	1	1	1	0	0	...	1	-2612.0	1	...
581	0	0	1	0	1	1	0	0	1	1	0	0	...	1	-2612.0	1	...
582	0	0	1	0	1	1	0	0	1	1	0	0	...	0	-2612.0	1	...
583	0	0	1	0	0	1	1	1	0	1	1	1	...	1	-2612.0	1	...
584	0	0	1	0	0	1	1	0	1	0	0	0	...	1	-2612.0	2	...
585	0	0	1	0	0	1	1	1	0	1	0	1	...	1	-2612.0	1	...
586	0	0	1	0	1	1	0	0	1	0	0	1	...	0	-2612.0	1	...
587	0	0	1	0	1	1	1	0	1	0	0	0	...	1	-2612.0	1	...
588	0	0	1	0	1	1	0	1	1	0	0	1	...	0	-2612.0	1	...
589	0	0	1	0	0	1	1	0	1	1	0	1	...	0	-2612.0	1	...
590	0	0	1	0	0	1	1	0	1	1	0	1	...	1	-2612.0	3	...
591	0	0	1	0	1	1	0	0	1	0	0	0	...	1	-2612.0	1	...
592	0	0	1	0	1	1	1	0	1	1	0	0	...	1	-2612.0	3	...
593	1	0	0	0	0	1	1	1	1	0	0	0	...	0	-2612.0	1	...
594	0	0	1	0	1	1	0	0	0	1	1	1	...	1	-2612.0	1	...
595	1	0	0	0	1	1	1	1	1	0	0	0	...	0	-2612.0	1	...
596	1	0	0	0	0	1	0	1	1	0	0	0	...	0	-2612.0	2	...
597	0	0	1	0	1	1	0	0	1	0	0	1	...	1	-2612.0	1	...
598	1	0	0	0	1	1	0	1	1	0	0	0	...	0	-2612.0	1	...
599	0	0	1	0	1	1	1	1	0	1	0	1	...	1	-2612.0	1	...
600	1	0	0	0	0	0	1	1	1	0	0	0	...	0	-2612.0	1	...
601	0	0	1	0	1	1	1	0	1	1	0	1	...	0	-2612.0	1	...
602	1	0	0	0	1	0	0	1	1	0	0	0	...	1	-2612.0	1	...
603	1	0	0	0	0	1	1	1	1	0	0	0	...	1	-2612.0	1	...
661	1	0	0	0	0	0	0	1	1	0	0	0	...	0	-2612.0	1	...
665	1	0	0	0	1	1	1	1	1	0	0	0	...	0	-2612.0	1	...

708	0	0	1	0	0	1	1	1	1	0	0	1	...	0	-2612.0	1	...
711	0	0	1	0	0	1	1	0	1	0	0	1	...	0	-2612.0	1	...
712	0	0	1	0	0	1	1	0	1	1	0	1	...	1	-2612.0	1	...
713	0	0	1	0	0	1	1	0	1	0	0	0	...	1	-2612.0	2	...
785	1	0	0	0	1	0	1	1	1	0	0	0	...	0	-2612.0	1	...
875	0	0	1	0	0	1	0	0	1	0	0	1	...	0	-2612.0	1	...
659	1	0	1	0	0	0	1	1	1	0	0	0	...	0	-2592.0	1	...
660	1	0	1	0	1	0	0	1	1	0	0	0	...	0	-2592.0	1	...
431	0	0	1	1	1	0	1	0	1	0	0	0	...	0	-2564.0	2	...
432	0	0	1	1	0	0	1	0	0	1	0	1	...	0	-2564.0	2	...
433	0	0	1	1	0	0	1	0	1	1	0	0	...	0	-2564.0	1	...
434	0	0	1	1	0	0	1	0	0	1	0	0	...	0	-2564.0	1	...
436	0	0	1	1	0	0	1	0	1	0	0	0	...	0	-2564.0	2	...
437	0	0	1	1	0	0	1	0	0	1	1	1	...	0	-2564.0	1	...
439	0	0	1	1	0	0	1	0	1	1	0	0	...	0	-2564.0	3	...
440	0	0	1	1	1	0	1	0	0	1	1	0	...	0	-2564.0	1	...
441	0	0	1	1	0	0	1	0	1	0	0	0	...	0	-2564.0	2	...
442	0	0	1	1	1	0	1	0	0	1	1	1	...	0	-2564.0	2	...
443	0	0	1	1	1	0	1	0	1	0	0	0	...	0	-2564.0	2	...
449	0	0	1	1	1	0	1	0	0	1	0	1	...	0	-2564.0	2	...
451	0	0	1	1	1	0	1	0	1	1	0	0	...	0	-2564.0	1	...
523	0	0	1	1	1	0	1	0	1	0	0	0	...	0	-2564.0	4	...
524	0	0	1	1	0	0	1	0	1	0	0	0	...	0	-2564.0	2	...
526	0	0	1	1	1	0	1	0	1	1	0	0	...	0	-2564.0	2	...
528	0	0	1	1	0	0	1	0	1	1	0	0	...	0	-2564.0	1	...
529	0	0	1	1	1	0	1	0	1	1	0	0	...	0	-2564.0	2	...
531	0	0	1	1	0	0	1	0	1	1	0	0	...	0	-2564.0	3	...
532	0	0	1	1	1	0	1	0	1	0	0	0	...	0	-2564.0	4	...
608	0	0	1	1	0	0	1	0	0	1	0	0	...	0	-2564.0	1	...
609	0	0	1	1	0	0	1	0	1	0	0	0	...	0	-2564.0	1	...
610	0	0	1	1	0	0	1	0	1	1	0	0	...	0	-2564.0	1	...
611	0	0	1	1	0	0	1	0	1	0	0	0	...	0	-2564.0	1	...
612	0	0	1	1	0	0	1	0	1	0	0	0	...	0	-2564.0	1	...
650	1	1	0	0	1	0	0	1	0	0	0	0	...	0	-2564.0	1	...
668	0	0	1	1	0	1	1	0	1	1	0	0	...	0	-2564.0	1	...
669	0	0	1	1	0	0	1	0	1	0	0	0	...	0	-2564.0	1	...
670	0	0	1	1	0	1	1	0	1	0	0	0	...	0	-2564.0	1	...
671	0	0	1	1	0	1	1	0	0	1	0	0	...	0	-2564.0	1	...
672	0	0	1	1	0	0	1	0	0	1	0	1	...	0	-2564.0	1	...
673	0	0	1	1	1	1	1	0	0	1	0	0	...	0	-2564.0	1	...
674	0	0	1	1	1	1	1	0	1	0	0	0	...	0	-2564.0	2	...
675	0	0	1	1	1	1	1	0	1	1	0	0	...	0	-2564.0	1	...
679	0	0	1	1	0	0	1	0	1	0	0	0	...	0	-2564.0	2	...
681	0	0	1	1	0	0	1	0	1	1	0	0	...	0	-2564.0	1	...

683	0	0	1	1	0	0	1	0	1	1	0	0	...	0	-2564.0	1	...
684	0	0	1	1	1	0	1	0	0	1	1	1	...	0	-2564.0	1	...
687	0	0	1	1	0	1	1	0	1	0	0	0	...	0	-2564.0	2	...
688	0	0	1	1	0	0	1	0	0	1	1	0	...	0	-2564.0	1	...
689	0	0	1	1	0	1	1	0	1	1	0	0	...	0	-2564.0	1	...
690	0	0	1	1	1	1	1	0	0	1	1	1	...	0	-2564.0	1	...
691	0	0	1	1	1	0	1	0	1	1	0	0	...	0	-2564.0	1	...
692	0	0	1	1	1	0	1	0	0	1	0	0	...	0	-2564.0	1	...
693	0	0	1	1	1	0	1	0	1	0	0	0	...	0	-2564.0	1	...
694	1	1	0	0	1	0	0	0	0	0	0	0	...	0	-2564.0	1	...
695	1	1	0	0	1	0	1	0	1	0	0	0	...	0	-2564.0	1	...
696	1	1	0	0	1	0	1	1	0	0	1	1	...	0	-2564.0	1	...
697	1	1	0	0	1	0	1	1	0	0	1	1	...	1	-2564.0	1	...
698	1	1	0	0	1	0	1	0	0	0	0	1	...	0	-2564.0	1	...
700	0	0	1	1	0	0	1	0	1	0	0	0	...	0	-2564.0	1	...
702	0	0	1	1	1	0	1	0	1	1	0	0	...	0	-2564.0	1	...
706	0	0	1	1	1	1	1	0	1	1	0	0	...	0	-2564.0	1	...
730	0	0	1	1	1	0	1	0	1	1	0	0	...	0	-2564.0	1	...
310	0	1	0	0	0	0	0	0	1	0	0	0	...	1	-2528.0	1	...
311	0	1	0	0	0	0	0	0	1	0	1	0	...	1	-2528.0	1	...
538	0	0	0	1	0	0	0	0	1	0	0	0	...	0	-2528.0	1	...
539	0	0	0	1	0	0	1	0	0	1	1	1	...	1	-2528.0	1	...
540	0	0	0	1	0	0	1	0	1	0	0	0	...	1	-2528.0	1	...
714	0	0	0	1	0	1	0	0	1	0	1	0	...	1	-2528.0	1	...
734	0	0	0	1	0	0	1	0	1	1	0	0	...	0	-2528.0	1	...
735	0	0	0	1	0	1	1	0	0	1	1	1	...	1	-2528.0	1	...
736	0	1	0	0	0	0	0	0	0	1	1	1	...	1	-2528.0	1	...
737	0	0	0	1	0	1	1	0	1	0	1	0	...	1	-2528.0	1	...
738	0	1	0	0	0	0	0	0	1	0	0	0	...	1	-2528.0	1	...
358	0	1	1	0	0	0	0	0	1	0	1	0	...	0	-2500.0	2	...
359	0	1	1	0	0	0	0	0	1	1	0	0	...	0	-2500.0	1	...
360	0	1	1	0	0	0	0	0	1	0	1	0	...	1	-2500.0	4	...
363	0	1	1	0	0	0	0	0	1	0	0	0	...	1	-2500.0	6	...
364	0	1	1	0	0	0	0	0	1	1	0	0	...	1	-2500.0	1	...
543	0	1	1	0	0	0	1	0	1	1	0	0	...	0	-2500.0	2	...
544	0	1	1	0	0	0	1	0	1	0	0	0	...	0	-2500.0	2	...
545	0	1	1	0	0	0	1	0	1	0	0	0	...	1	-2500.0	5	...
546	0	1	1	0	0	0	1	0	1	0	1	0	...	1	-2500.0	5	...
619	0	1	1	0	0	0	1	0	1	0	0	0	...	0	-2500.0	1	...
621	1	0	0	1	0	0	0	1	0	0	0	1	...	0	-2500.0	1	...
622	1	0	0	1	0	0	0	1	0	0	1	1	...	0	-2500.0	1	...
623	0	1	1	0	0	1	0	0	1	1	0	0	...	0	-2500.0	1	...
626	1	0	0	1	1	0	0	1	0	1	0	0	...	0	-2500.0	1	...
634	1	0	0	1	0	0	0	1	0	1	1	1	...	0	-2500.0	1	...

638	1	0	0	1	1	0	0	1	0	0	1	0	...	0	-2500.0	1	...
641	1	0	0	1	0	0	0	1	0	0	1	1	...	0	-2500.0	2	...
643	0	1	1	0	0	1	1	0	1	0	0	0	...	0	-2500.0	2	...
644	0	1	1	0	0	1	1	0	1	0	0	0	...	1	-2500.0	2	...
647	1	0	0	1	1	0	0	1	0	0	0	1	...	0	-2500.0	1	...
648	1	0	0	1	1	0	0	1	0	1	0	1	...	0	-2500.0	1	...
649	0	1	1	0	0	0	1	0	1	0	0	0	...	1	-2500.0	1	...
651	0	1	1	0	0	1	0	0	1	0	0	0	...	1	-2500.0	2	...
652	0	1	1	0	0	0	0	0	1	1	0	0	...	1	-2500.0	1	...
741	0	1	1	0	0	0	1	0	1	1	0	0	...	0	-2500.0	1	...
743	1	0	0	1	1	0	0	1	0	0	1	0	...	0	-2500.0	1	...
750	0	1	1	0	0	0	0	0	1	0	1	0	...	1	-2500.0	1	...
751	0	1	1	0	0	0	0	0	1	0	1	1	...	0	-2500.0	1	...
752	0	1	1	0	0	0	1	0	1	0	1	1	...	0	-2500.0	1	...
753	0	1	1	0	0	0	0	0	1	1	0	1	...	1	-2500.0	1	...
754	0	1	1	0	0	0	0	0	1	0	0	1	...	1	-2500.0	1	...
755	1	0	0	1	1	0	0	1	0	1	1	0	...	1	-2500.0	1	...
756	0	1	1	0	0	1	1	0	1	0	1	1	...	1	-2500.0	1	...
757	0	1	1	0	0	0	0	0	1	0	1	1	...	1	-2500.0	1	...
758	0	1	1	0	0	0	1	0	1	0	1	0	...	1	-2500.0	1	...
759	0	1	1	0	0	0	1	0	1	1	0	0	...	1	-2500.0	1	...
760	0	1	1	0	0	1	1	0	1	0	0	0	...	1	-2500.0	1	...
761	0	1	1	0	0	1	1	0	1	0	0	1	...	1	-2500.0	2	...
762	0	1	1	0	0	0	1	0	1	1	0	1	...	1	-2500.0	1	...
771	0	1	1	0	0	0	1	0	1	0	1	0	...	1	-2500.0	1	...
840	0	1	1	0	0	0	1	0	1	0	0	1	...	1	-2500.0	1	...
796	1	0	1	0	0	0	0	0	0	1	1	0	...	0	-2464.0	1	...
345	0	0	1	1	0	0	0	0	1	0	0	0	...	1	-2436.0	11	...
346	0	0	1	1	0	0	0	0	1	1	0	0	...	1	-2436.0	8	...
347	0	0	1	1	0	0	0	0	1	1	0	0	...	1	-2436.0	12	...
350	0	0	1	1	0	0	0	0	1	0	0	0	...	1	-2436.0	3	...
354	0	0	1	1	1	0	0	0	1	0	0	0	...	1	-2436.0	12	...
355	0	0	1	1	1	0	0	0	1	1	0	0	...	1	-2436.0	8	...
356	0	0	1	1	1	0	0	0	1	0	0	0	...	1	-2436.0	3	...
357	0	0	1	1	1	0	0	0	1	1	0	0	...	1	-2436.0	7	...
444	1	1	0	0	0	0	0	1	0	0	1	0	...	0	-2436.0	2	...
446	1	1	0	0	0	0	1	1	0	0	1	0	...	1	-2436.0	1	...
447	1	1	0	0	0	0	0	1	1	0	0	0	...	0	-2436.0	1	...
452	1	1	0	0	0	0	1	1	0	0	1	1	...	0	-2436.0	1	...
453	1	1	0	0	0	0	0	1	0	0	1	1	...	0	-2436.0	1	...
455	1	1	0	0	0	0	0	1	0	0	0	1	...	0	-2436.0	6	...
456	1	1	0	0	0	0	0	1	0	0	0	0	...	1	-2436.0	1	...
457	1	1	0	0	0	0	1	1	0	0	1	1	...	1	-2436.0	1	...
459	1	1	0	0	0	0	1	1	0	0	0	1	...	0	-2436.0	1	...

463	1	1	0	0	0	0	1	1	0	0	0	1	...	1	-2436.0	4	...
468	1	1	0	0	0	0	1	1	0	0	1	1	...	0	-2436.0	1	...
478	1	1	0	0	0	0	0	1	0	0	1	1	...	0	-2436.0	2	...
480	1	1	0	0	0	0	1	1	0	0	0	0	...	0	-2436.0	1	...
487	1	1	0	0	0	0	1	1	0	0	0	1	...	1	-2436.0	1	...
516	0	0	1	1	0	0	0	0	1	1	0	0	...	1	-2436.0	2	...
517	0	0	1	1	0	0	0	0	1	0	0	0	...	1	-2436.0	1	...
518	0	0	1	1	0	0	0	0	1	0	0	0	...	1	-2436.0	1	...
519	0	0	1	1	0	0	0	0	1	0	0	0	...	1	-2436.0	2	...
520	0	0	1	1	0	0	0	0	1	1	0	0	...	1	-2436.0	2	...
521	0	0	1	1	0	0	0	0	1	1	0	0	...	1	-2436.0	1	...
522	0	0	1	1	0	0	0	0	1	1	0	0	...	1	-2436.0	1	...
567	0	0	1	1	0	1	0	0	1	0	0	0	...	1	-2436.0	3	...
569	0	0	1	1	0	1	0	0	1	1	0	0	...	1	-2436.0	2	...
571	0	0	1	1	0	1	0	0	1	1	0	0	...	1	-2436.0	2	...
573	0	0	1	1	0	1	0	0	1	0	0	0	...	1	-2436.0	1	...
574	0	0	1	1	0	0	0	0	1	1	0	0	...	1	-2436.0	3	...
575	0	0	1	1	1	1	0	0	1	0	0	0	...	1	-2436.0	1	...
576	0	0	1	1	1	1	0	0	1	1	0	0	...	1	-2436.0	2	...
577	0	0	1	1	1	1	0	0	1	0	0	0	...	1	-2436.0	1	...
578	0	0	1	1	1	1	0	0	1	1	0	0	...	1	-2436.0	2	...
604	0	0	1	1	0	0	0	0	1	1	0	0	...	1	-2436.0	1	...
605	0	0	1	1	1	0	0	0	1	0	0	0	...	1	-2436.0	1	...
606	0	0	1	1	1	0	0	0	1	0	0	0	...	1	-2436.0	1	...
607	0	0	1	1	0	0	0	0	1	1	0	0	...	1	-2436.0	2	...
614	0	0	1	1	0	0	0	0	1	1	0	0	...	1	-2436.0	2	...
615	0	0	1	1	0	0	0	0	1	0	0	0	...	1	-2436.0	2	...
616	0	0	1	1	0	0	0	0	1	1	0	0	...	1	-2436.0	1	...
618	0	0	1	1	0	1	0	0	1	1	0	0	...	1	-2436.0	3	...
620	0	0	1	1	1	1	0	0	1	0	0	0	...	1	-2436.0	1	...
624	0	0	1	1	1	1	0	0	1	0	0	0	...	1	-2436.0	1	...
627	0	0	1	1	0	1	0	0	1	0	0	0	...	1	-2436.0	1	...
629	0	0	1	1	1	0	0	0	1	1	0	0	...	1	-2436.0	1	...
630	0	0	1	1	1	1	0	0	1	1	0	0	...	1	-2436.0	2	...
631	0	0	1	1	1	1	0	0	1	1	0	0	...	1	-2436.0	1	...
632	0	0	1	1	1	0	0	0	1	1	0	0	...	1	-2436.0	1	...
635	0	0	1	1	1	0	0	0	1	0	0	0	...	1	-2436.0	1	...
636	0	0	1	1	1	1	0	0	1	0	0	0	...	1	-2436.0	1	...
637	0	0	1	1	1	0	0	0	1	1	0	0	...	1	-2436.0	2	...
639	0	0	1	1	0	1	0	0	1	1	0	0	...	1	-2436.0	1	...
640	0	0	1	1	1	0	0	0	1	0	0	0	...	1	-2436.0	2	...
715	0	0	1	1	0	0	0	0	1	1	0	0	...	1	-2436.0	1	...
716	0	0	1	1	0	0	0	0	1	1	0	0	...	1	-2436.0	1	...
717	0	0	1	1	0	0	0	0	1	0	0	0	...	1	-2436.0	1	...

721	0	0	1	1	0	0	0	0	0	0	1	0	...	0	-2436.0	1	...
722	0	0	1	1	0	0	0	0	1	0	1	0	...	0	-2436.0	1	...
723	0	0	1	1	1	0	0	0	1	0	0	1	...	0	-2436.0	1	...
724	0	0	1	1	1	0	0	0	0	0	0	0	...	0	-2436.0	1	...
725	0	0	1	1	0	0	0	0	1	1	0	1	...	0	-2436.0	2	...
726	0	0	1	1	0	0	0	0	1	1	1	0	...	0	-2436.0	1	...
727	0	0	1	1	0	0	0	0	1	0	0	1	...	0	-2436.0	1	...
728	0	0	1	1	0	0	0	0	1	1	0	0	...	1	-2436.0	1	...
739	0	0	1	1	1	1	0	0	1	0	0	0	...	1	-2436.0	1	...
744	0	0	1	1	0	0	0	0	1	1	0	0	...	1	-2436.0	1	...
800	0	0	1	1	1	1	0	0	1	0	0	1	...	0	-2436.0	1	...
803	0	0	1	1	0	0	0	0	0	1	1	1	...	1	-2436.0	1	...
813	0	0	1	1	1	0	0	0	1	0	0	0	...	1	-2436.0	1	...
815	0	0	1	1	0	0	0	0	1	0	1	0	...	0	-2436.0	1	...
816	0	0	1	1	1	0	0	0	1	0	1	0	...	0	-2436.0	1	...
817	1	1	0	0	0	0	1	1	1	0	0	0	...	0	-2436.0	1	...
818	0	0	1	1	0	0	0	0	1	1	0	0	...	1	-2436.0	1	...
819	1	1	0	0	0	0	1	1	0	1	1	1	...	0	-2436.0	1	...
820	1	1	0	0	0	0	1	0	0	0	1	0	...	0	-2436.0	1	...
821	1	1	0	0	0	0	0	0	0	0	1	1	...	0	-2436.0	1	...
822	0	0	1	1	0	1	0	0	0	1	0	1	...	1	-2436.0	1	...
829	1	1	0	0	0	0	0	1	0	0	0	1	...	0	-2436.0	1	...
831	1	1	0	0	0	0	1	1	0	0	1	1	...	1	-2436.0	1	...
839	1	1	0	0	0	0	0	1	0	0	0	1	...	1	-2436.0	1	...
842	0	0	1	1	0	0	0	0	1	0	1	0	...	0	-2436.0	1	...
843	0	0	1	1	0	0	0	0	1	0	0	1	...	0	-2436.0	1	...
882	0	0	1	1	1	0	0	0	0	1	0	1	...	1	-2436.0	1	...
823	0	0	0	0	0	1	1	1	1	0	1	1	...	1	-2432.0	1	...
825	0	0	0	1	1	1	1	0	1	0	1	0	...	0	-2400.0	1	...
826	0	0	0	1	1	0	1	0	0	1	0	1	...	1	-2400.0	1	...
833	0	0	0	1	1	0	1	1	1	0	0	1	...	0	-2400.0	1	...
834	0	1	0	0	0	0	1	0	0	0	1	1	...	0	-2400.0	1	...
835	0	1	0	0	0	1	1	0	0	1	1	0	...	1	-2400.0	1	...
685	0	1	1	0	1	0	0	0	1	0	1	0	...	0	-2372.0	1	...
686	0	1	1	0	1	0	0	0	0	0	0	0	...	1	-2372.0	1	...
789	1	0	0	1	0	0	1	1	0	0	0	1	...	0	-2372.0	2	...
791	1	0	0	1	1	0	1	1	0	1	0	0	...	0	-2372.0	1	...
794	1	0	0	1	1	0	1	1	0	0	1	1	...	0	-2372.0	1	...
795	0	1	1	0	1	1	1	0	1	0	1	0	...	1	-2372.0	1	...
841	0	1	1	0	1	0	1	0	1	1	0	1	...	1	-2372.0	1	...
185	1	0	0	0	0	0	0	1	0	0	1	0	...	0	-2356.0	1	...
186	1	0	0	0	0	0	0	1	0	0	1	0	...	0	-2356.0	4	...
187	1	0	0	0	0	1	0	1	0	0	1	0	...	0	-2356.0	5	...
188	1	0	0	0	0	1	0	1	0	0	1	0	...	0	-2356.0	5	...

189	1	0	0	0	0	0	1	1	0	0	1	0	...	0	-2356.0	2	...
190	1	0	0	0	0	0	1	1	0	0	1	0	...	0	-2356.0	4	...
191	1	0	0	0	0	0	0	1	0	0	1	1	...	0	-2356.0	3	...
192	1	0	0	0	0	0	1	1	0	0	0	0	...	0	-2356.0	4	...
193	1	0	0	0	0	1	0	1	0	0	0	0	...	0	-2356.0	1	...
194	1	0	0	0	0	0	0	1	0	0	0	1	...	0	-2356.0	16	...
195	1	0	0	0	0	1	1	1	0	0	1	0	...	0	-2356.0	4	...
196	1	0	0	0	1	1	0	1	0	0	1	1	...	0	-2356.0	4	...
197	1	0	0	0	0	0	1	1	0	0	1	1	...	0	-2356.0	5	...
198	1	0	0	0	0	1	1	1	0	0	1	1	...	0	-2356.0	10	...
199	1	0	0	0	0	1	1	1	0	0	1	0	...	0	-2356.0	6	...
200	1	0	0	0	0	0	0	1	0	0	0	0	...	0	-2356.0	1	...
202	1	0	0	0	0	0	1	1	0	0	1	1	...	0	-2356.0	14	...
205	1	0	0	0	1	0	0	1	0	0	1	0	...	0	-2356.0	1	...
206	1	0	0	0	1	0	0	1	0	0	1	1	...	0	-2356.0	1	...
207	1	0	0	0	1	0	0	1	0	0	1	0	...	0	-2356.0	1	...
208	1	0	0	0	1	0	1	1	0	0	1	0	...	0	-2356.0	1	...
209	1	0	0	0	0	1	0	1	0	0	1	1	...	0	-2356.0	6	...
210	1	0	0	0	1	0	1	1	0	0	1	0	...	0	-2356.0	3	...
211	1	0	0	0	0	1	0	1	0	0	1	1	...	0	-2356.0	11	...
212	1	0	0	0	0	0	1	1	0	0	0	0	...	0	-2356.0	2	...
213	1	0	0	0	0	0	0	1	0	0	0	0	...	0	-2356.0	3	...
214	1	0	0	0	0	0	0	1	0	0	1	1	...	0	-2356.0	5	...
215	1	0	0	0	1	1	0	1	0	0	0	0	...	0	-2356.0	1	...
216	1	0	0	0	1	0	0	1	0	0	0	1	...	0	-2356.0	1	...
217	1	0	0	0	0	1	0	1	0	0	0	0	...	1	-2356.0	1	...
219	1	0	0	0	0	0	1	1	0	0	0	1	...	0	-2356.0	10	...
221	1	0	0	0	1	1	0	1	0	0	1	1	...	1	-2356.0	1	...
222	1	0	0	0	0	1	0	1	0	0	1	0	...	1	-2356.0	1	...
223	1	0	0	0	0	1	0	1	0	0	1	1	...	1	-2356.0	2	...
224	1	0	0	0	0	0	1	1	0	0	0	1	...	0	-2356.0	16	...
225	1	0	0	0	0	1	1	1	0	0	0	0	...	0	-2356.0	3	...
226	1	0	0	0	1	0	0	1	0	0	1	1	...	0	-2356.0	5	...
228	1	0	0	0	1	1	0	1	0	0	0	0	...	0	-2356.0	2	...
229	1	0	0	0	1	1	1	1	0	0	0	0	...	0	-2356.0	5	...
230	1	0	0	0	0	1	1	1	0	0	1	1	...	0	-2356.0	10	...
231	1	0	0	0	0	0	1	1	0	0	1	1	...	1	-2356.0	6	...
232	1	0	0	0	0	1	1	1	0	0	1	0	...	1	-2356.0	1	...
235	1	0	0	0	1	1	1	1	0	0	1	1	...	0	-2356.0	4	...
236	1	0	0	0	0	1	1	1	0	0	1	1	...	1	-2356.0	4	...
237	1	0	0	0	0	1	1	1	0	0	0	1	...	0	-2356.0	12	...
238	1	0	0	0	1	0	1	1	0	0	1	1	...	0	-2356.0	7	...
239	1	0	0	0	1	1	1	1	0	0	1	0	...	0	-2356.0	3	...
240	1	0	0	0	0	0	0	1	0	0	1	1	...	1	-2356.0	1	...

241	1	0	0	0	0	1	1	1	0	0	0	1	...	0	-2356.0	16	...
242	1	0	0	0	0	1	1	1	0	0	0	0	...	0	-2356.0	2	...
243	1	0	0	0	0	0	0	1	0	0	0	1	...	0	-2356.0	4	...
246	1	0	0	0	1	1	1	1	0	0	1	1	...	0	-2356.0	3	...
247	1	0	0	0	0	0	0	1	0	0	0	0	...	1	-2356.0	2	...
248	1	0	0	0	0	1	0	1	0	0	0	1	...	0	-2356.0	10	...
249	1	0	0	0	0	0	0	1	0	0	0	0	...	1	-2356.0	4	...
250	1	0	0	0	1	0	0	1	0	0	1	1	...	1	-2356.0	1	...
251	1	0	0	0	0	1	0	1	0	0	1	1	...	1	-2356.0	2	...
252	1	0	0	0	0	0	1	1	0	0	1	0	...	1	-2356.0	2	...
254	1	0	0	0	1	1	0	1	0	0	1	1	...	0	-2356.0	4	...
255	1	0	0	0	1	1	0	1	0	0	1	0	...	1	-2356.0	1	...
256	1	0	0	0	0	0	0	1	0	0	1	1	...	1	-2356.0	6	...
257	1	0	0	0	0	1	0	1	0	0	0	1	...	0	-2356.0	9	...
259	1	0	0	0	1	0	0	1	0	0	1	0	...	1	-2356.0	1	...
260	1	0	0	0	1	0	0	1	0	0	0	0	...	0	-2356.0	1	...
261	1	0	0	0	0	0	1	1	0	0	1	1	...	1	-2356.0	2	...
262	1	0	0	0	1	1	0	1	0	0	0	1	...	0	-2356.0	9	...
263	1	0	0	0	0	0	0	1	0	0	0	1	...	1	-2356.0	4	...
265	1	0	0	0	1	1	1	1	0	0	1	1	...	1	-2356.0	1	...
267	1	0	0	0	0	1	1	1	0	0	0	0	...	1	-2356.0	1	...
268	1	0	0	0	1	0	0	1	0	0	0	0	...	1	-2356.0	1	...
269	1	0	0	0	0	0	0	1	0	0	0	1	...	1	-2356.0	1	...
270	1	0	0	0	0	1	0	1	0	0	0	1	...	1	-2356.0	1	...
271	1	0	0	0	1	1	1	1	0	0	0	0	...	1	-2356.0	2	...
272	1	0	0	0	1	0	0	1	0	0	1	1	...	1	-2356.0	1	...
273	1	0	0	0	1	0	0	1	0	0	0	1	...	0	-2356.0	5	...
274	1	0	0	0	0	1	1	1	0	0	0	1	...	1	-2356.0	4	...
275	1	0	0	0	1	0	1	1	0	0	1	1	...	1	-2356.0	1	...
276	1	0	0	0	1	0	1	1	0	0	0	1	...	0	-2356.0	5	...
277	1	0	0	0	0	1	1	1	0	0	0	1	...	1	-2356.0	3	...
278	1	0	0	0	0	1	1	1	0	0	0	0	...	1	-2356.0	3	...
279	1	0	0	0	1	1	1	1	0	0	0	1	...	0	-2356.0	7	...
280	1	0	0	0	1	1	0	1	0	0	0	1	...	1	-2356.0	1	...
281	1	0	0	0	0	0	1	1	0	0	0	1	...	1	-2356.0	2	...
282	1	0	0	0	1	1	1	1	0	0	0	1	...	0	-2356.0	8	...
283	1	0	0	0	1	1	0	1	0	0	0	0	...	1	-2356.0	1	...
285	1	0	0	0	1	1	1	1	0	0	0	0	...	1	-2356.0	1	...
287	1	0	0	0	0	1	1	1	0	0	1	1	...	1	-2356.0	4	...
288	1	0	0	0	1	1	0	1	0	0	1	1	...	1	-2356.0	3	...
289	1	0	0	0	1	0	1	1	0	0	0	1	...	0	-2356.0	3	...
290	1	0	0	0	1	0	1	1	0	0	1	1	...	1	-2356.0	1	...
291	1	0	0	0	1	1	0	1	0	0	0	1	...	0	-2356.0	2	...
292	1	0	0	0	0	0	1	1	0	0	0	1	...	1	-2356.0	3	...

293	1	0	0	0	0	1	0	1	0	0	0	1	...	1	-2356.0	5	...
294	1	0	0	0	1	1	1	1	0	0	0	1	...	1	-2356.0	6	...
295	1	0	0	0	1	0	1	1	0	0	0	1	...	1	-2356.0	2	...
296	1	0	0	0	1	0	1	1	0	0	0	1	...	1	-2356.0	2	...
297	1	0	0	0	1	1	0	1	0	0	0	1	...	1	-2356.0	3	...
298	1	0	0	0	1	0	0	1	0	0	0	1	...	1	-2356.0	3	...
299	1	0	0	0	1	1	1	1	0	0	0	1	...	1	-2356.0	3	...
300	0	1	1	1	0	0	0	0	0	1	0	1	...	0	-2356.0	1	...
420	0	0	1	0	0	1	0	0	1	0	0	1	...	0	-2356.0	1	...
542	0	1	1	1	0	0	0	0	1	0	0	0	...	0	-2356.0	1	...
662	1	0	0	0	0	0	0	1	0	1	0	1	...	0	-2356.0	1	...
663	1	0	0	0	0	0	0	1	1	0	0	1	...	0	-2356.0	1	...
664	1	0	0	0	0	0	0	1	1	0	0	1	...	0	-2356.0	1	...
666	1	0	0	0	0	1	0	1	0	1	0	1	...	1	-2356.0	1	...
667	1	0	0	0	0	1	0	1	0	0	0	1	...	1	-2356.0	1	...
709	0	0	1	0	0	1	1	0	1	0	0	0	...	0	-2356.0	1	...
718	1	0	0	0	0	0	0	1	0	0	0	1	...	0	-2356.0	1	...
719	1	0	0	0	0	1	0	1	0	0	0	1	...	0	-2356.0	1	...
720	1	0	0	0	1	1	1	1	0	0	0	1	...	0	-2356.0	1	...
764	1	0	0	0	0	1	1	1	1	0	0	1	...	0	-2356.0	2	...
765	1	0	0	0	0	1	1	1	1	0	0	1	...	0	-2356.0	1	...
766	1	0	0	0	0	1	0	1	0	1	1	1	...	0	-2356.0	1	...
767	1	0	0	0	0	0	1	1	0	0	1	0	...	1	-2356.0	1	...
768	1	0	0	0	0	1	1	1	0	0	1	0	...	1	-2356.0	1	...
769	1	0	0	0	0	0	0	1	0	0	1	1	...	1	-2356.0	1	...
770	1	0	0	0	0	1	1	1	0	0	0	1	...	1	-2356.0	1	...
773	1	0	0	0	0	1	1	1	0	0	1	1	...	0	-2356.0	1	...
774	1	0	0	0	0	1	1	1	0	0	1	0	...	0	-2356.0	1	...
775	1	0	0	0	0	0	1	1	0	0	1	0	...	0	-2356.0	1	...
776	1	0	0	0	1	0	1	1	0	0	1	1	...	0	-2356.0	1	...
777	1	0	0	0	0	0	1	1	0	0	0	1	...	1	-2356.0	1	...
778	1	0	0	0	0	1	0	1	0	0	0	0	...	1	-2356.0	1	...
779	1	0	0	0	0	1	0	1	0	0	0	1	...	1	-2356.0	1	...
780	1	0	0	0	1	1	0	1	0	1	0	1	...	0	-2356.0	1	...
781	1	0	0	0	0	1	1	1	0	0	1	1	...	1	-2356.0	1	...
782	1	0	0	0	1	1	1	1	0	1	1	0	...	0	-2356.0	1	...
783	1	0	0	0	1	0	1	1	0	0	0	0	...	0	-2356.0	1	...
784	1	0	0	0	0	0	1	1	0	0	1	1	...	1	-2356.0	2	...
786	1	0	0	0	1	0	1	1	0	0	0	1	...	1	-2356.0	1	...
787	0	0	1	0	1	1	1	0	1	0	0	0	...	1	-2356.0	1	...
797	1	0	0	0	0	0	1	1	0	1	0	0	...	0	-2356.0	1	...
798	1	0	0	0	1	1	0	1	0	1	0	1	...	0	-2356.0	1	...
799	1	0	0	0	1	0	0	1	1	0	0	1	...	0	-2356.0	1	...
801	0	0	1	0	1	1	1	0	1	1	0	0	...	1	-2356.0	1	...

810	1	0	0	0	0	1	1	1	0	0	0	1	...	0	-2356.0	1	...
811	1	0	0	0	0	1	1	1	0	0	0	1	...	0	-2356.0	1	...
812	1	0	0	0	1	1	1	1	0	0	0	1	...	1	-2356.0	1	...
824	0	0	1	0	0	1	0	1	1	0	0	1	...	0	-2356.0	1	...
838	0	0	1	0	1	1	0	0	1	1	0	1	...	1	-2356.0	1	...
845	0	0	1	0	0	1	1	0	1	0	1	0	...	0	-2356.0	1	...
846	1	0	0	0	0	1	0	1	0	1	1	0	...	0	-2356.0	1	...
847	1	0	0	0	0	1	1	1	0	0	0	1	...	0	-2356.0	1	...
848	1	0	0	0	1	0	1	1	0	0	0	1	...	0	-2356.0	1	...
849	1	0	0	0	1	1	1	1	0	1	0	0	...	1	-2356.0	1	...
869	1	0	0	0	0	0	1	1	0	0	1	1	...	0	-2356.0	1	...
873	0	0	1	0	1	1	1	0	1	0	0	0	...	1	-2356.0	1	...
877	1	0	0	0	0	0	1	1	0	0	0	1	...	0	-2356.0	1	...
879	1	0	0	0	1	1	1	1	0	0	0	1	...	0	-2356.0	1	...
855	1	0	1	0	0	1	1	0	1	0	0	0	...	0	-2336.0	1	...
464	1	1	0	0	0	1	0	1	0	0	1	1	...	0	-2308.0	1	...
465	1	1	0	0	0	1	1	1	0	0	1	0	...	0	-2308.0	1	...
467	1	1	0	0	0	1	0	1	0	0	1	0	...	0	-2308.0	1	...
474	1	1	0	0	0	1	1	1	0	0	0	1	...	0	-2308.0	1	...
475	1	1	0	0	0	1	0	1	0	0	0	1	...	0	-2308.0	2	...
481	1	1	0	0	0	1	1	1	0	0	0	0	...	0	-2308.0	1	...
486	1	1	0	0	0	1	0	1	0	0	0	1	...	1	-2308.0	1	...
488	1	1	0	0	0	1	1	1	0	0	0	1	...	1	-2308.0	2	...
525	0	0	1	1	0	0	1	0	1	0	0	0	...	1	-2308.0	4	...
527	0	0	1	1	0	0	1	0	1	1	0	0	...	1	-2308.0	7	...
530	0	0	1	1	0	0	1	0	1	1	0	0	...	1	-2308.0	3	...
533	0	0	1	1	1	0	1	0	1	1	0	0	...	1	-2308.0	4	...
534	0	0	1	1	1	0	1	0	1	0	0	0	...	1	-2308.0	3	...
535	0	0	1	1	1	0	1	0	1	1	0	0	...	1	-2308.0	5	...
536	0	0	1	1	1	0	1	0	1	0	0	0	...	1	-2308.0	3	...
653	1	1	0	0	1	0	1	1	0	0	1	0	...	0	-2308.0	1	...
677	0	0	1	1	0	0	1	0	1	0	0	0	...	1	-2308.0	2	...
678	0	0	1	1	0	0	1	0	1	1	0	0	...	1	-2308.0	2	...
680	0	0	1	1	0	0	1	0	1	0	0	0	...	1	-2308.0	1	...
682	0	0	1	1	0	0	1	0	1	1	0	0	...	1	-2308.0	1	...
699	0	0	1	1	0	0	1	0	1	1	0	0	...	1	-2308.0	2	...
701	0	0	1	1	0	1	1	0	1	1	0	0	...	1	-2308.0	1	...
703	0	0	1	1	0	0	1	0	1	0	0	0	...	1	-2308.0	1	...
704	0	0	1	1	0	1	1	0	1	0	0	0	...	1	-2308.0	1	...
705	0	0	1	1	1	1	1	0	1	0	0	0	...	1	-2308.0	2	...
707	0	0	1	1	1	1	1	0	1	1	0	0	...	1	-2308.0	1	...
729	1	1	0	0	1	0	0	1	0	0	1	1	...	0	-2308.0	1	...
732	0	0	1	1	1	0	1	0	1	1	0	0	...	1	-2308.0	1	...
733	0	0	1	1	1	1	1	0	1	1	0	0	...	1	-2308.0	1	...

772	0	0	1	1	1	0	1	0	1	0	0	0	...	1	-2308.0	1 ...
827	0	0	1	1	0	0	1	0	1	0	0	1	...	0	-2308.0	1 ...
828	0	0	1	1	1	0	1	0	0	1	0	0	...	1	-2308.0	1 ...
837	0	0	1	1	0	0	1	0	0	1	0	1	...	1	-2308.0	1 ...
850	0	0	1	1	0	0	1	0	0	1	0	0	...	1	-2308.0	1 ...
857	0	0	1	1	0	1	0	1	1	1	0	0	...	0	-2308.0	1 ...
858	1	1	0	0	0	1	0	0	0	0	0	1	...	0	-2308.0	1 ...
862	0	0	1	1	1	1	1	0	1	1	0	0	...	1	-2308.0	1 ...
881	0	0	1	1	1	0	1	0	1	0	0	0	...	1	-2308.0	1 ...
872	0	0	0	0	1	1	1	1	1	0	1	1	...	1	-2304.0	1 ...
863	0	1	0	0	0	1	0	1	0	0	1	0	...	1	-2272.0	1 ...
625	1	0	0	1	0	0	0	1	0	0	0	1	...	0	-2244.0	1 ...
642	1	0	0	1	0	0	0	1	0	1	0	1	...	0	-2244.0	1 ...
645	1	0	0	1	1	0	0	1	0	0	0	1	...	0	-2244.0	2 ...
646	1	0	0	1	1	0	0	1	0	0	0	1	...	0	-2244.0	1 ...
654	1	0	0	1	1	0	0	1	0	1	0	1	...	0	-2244.0	1 ...
748	1	0	0	1	1	0	0	1	0	0	1	1	...	0	-2244.0	1 ...
854	0	1	1	0	0	1	1	0	0	1	0	0	...	1	-2244.0	1 ...
865	0	1	1	0	1	0	1	1	1	0	0	1	...	0	-2244.0	1 ...
2	0	0	1	0	0	0	0	1	1	0	0	0	...	1	-2228.0	2 ...
4	0	0	1	0	0	0	0	1	1	0	0	0	...	0	-2228.0	4 ...
5	0	0	1	0	0	0	0	0	1	0	0	0	...	0	-2228.0	2 ...
8	0	0	1	0	0	0	0	0	1	0	0	1	...	0	-2228.0	2 ...
9	0	0	1	0	0	0	0	0	1	0	0	0	...	1	-2228.0	3 ...
11	0	0	1	0	0	0	0	0	1	0	0	1	...	1	-2228.0	4 ...
12	0	0	1	0	0	0	0	1	1	0	0	1	...	0	-2228.0	2 ...
14	0	0	1	0	0	0	0	1	1	0	0	1	...	1	-2228.0	3 ...
41	0	0	1	0	0	0	1	1	1	0	0	0	...	0	-2228.0	2 ...
42	0	0	1	0	0	0	1	1	1	0	0	0	...	1	-2228.0	1 ...
43	0	0	1	0	0	0	1	0	1	0	0	0	...	1	-2228.0	1 ...
46	0	0	1	0	0	0	1	0	1	0	0	1	...	0	-2228.0	1 ...
49	0	0	1	0	1	0	0	0	1	0	0	0	...	1	-2228.0	3 ...
50	0	0	1	0	0	0	1	0	1	0	0	1	...	1	-2228.0	1 ...
56	0	0	1	0	1	0	0	1	1	0	0	1	...	1	-2228.0	1 ...
417	0	0	1	0	0	0	0	0	1	0	0	0	...	1	-2228.0	1 ...
419	0	0	1	0	0	0	0	1	1	0	0	0	...	0	-2228.0	1 ...
422	0	0	1	0	1	0	1	0	1	0	0	0	...	1	-2228.0	1 ...
423	0	0	1	0	1	0	1	1	1	0	0	1	...	0	-2228.0	3 ...
470	0	1	1	1	1	0	0	0	1	0	0	0	...	0	-2228.0	1 ...
617	0	1	1	1	0	0	1	0	1	0	0	0	...	0	-2228.0	1 ...
710	0	0	1	0	0	0	1	1	0	1	0	1	...	0	-2228.0	1 ...
658	1	1	0	0	1	1	1	1	0	0	0	1	...	1	-2180.0	1 ...
802	0	0	1	1	1	0	0	0	1	0	1	0	...	1	-2180.0	1 ...
804	0	0	1	1	0	0	0	0	1	1	0	1	...	1	-2180.0	1 ...

805	0	0	1	1	0	0	0	0	1	0	0	1	...	1	-2180.0	1	...
806	0	0	1	1	0	0	0	0	1	0	0	1	...	1	-2180.0	2	...
807	0	0	1	1	1	0	0	0	1	1	0	1	...	1	-2180.0	1	...
808	0	0	1	1	1	0	0	0	1	0	0	1	...	1	-2180.0	1	...
809	0	0	1	1	1	0	0	0	1	1	0	1	...	1	-2180.0	1	...
844	0	0	1	1	1	1	0	0	1	0	1	0	...	1	-2180.0	1	...
853	0	0	1	1	0	1	0	0	1	1	0	1	...	1	-2180.0	1	...
870	0	0	1	1	0	0	1	1	1	1	0	0	...	0	-2180.0	1	...
860	0	0	0	0	0	1	1	1	1	0	0	1	...	1	-2176.0	1	...
745	1	0	0	1	0	1	0	1	0	1	0	1	...	0	-2116.0	1	...
747	1	0	0	1	1	1	0	1	1	0	0	0	...	0	-2116.0	1	...
790	1	0	0	1	0	0	1	1	0	0	0	1	...	0	-2116.0	1	...
792	1	0	0	1	1	0	1	1	0	0	0	1	...	0	-2116.0	1	...
793	1	0	0	1	1	0	1	1	0	0	1	1	...	0	-2116.0	1	...
814	1	0	0	1	1	0	1	1	0	0	0	1	...	0	-2116.0	1	...
302	0	1	1	1	0	0	0	0	0	1	1	1	...	0	-2100.0	1	...
859	1	1	1	0	0	1	0	0	0	0	1	1	...	0	-2080.0	1	...
469	1	1	0	0	0	1	1	1	0	0	1	0	...	0	-2052.0	1	...
471	1	1	0	0	0	1	0	1	0	0	1	0	...	0	-2052.0	1	...
476	1	1	0	0	0	1	1	1	0	0	1	1	...	0	-2052.0	1	...
477	1	1	0	0	0	1	0	1	0	0	1	0	...	1	-2052.0	1	...
479	1	1	0	0	0	1	1	1	0	0	0	1	...	0	-2052.0	2	...
482	1	1	0	0	0	1	1	1	0	0	0	0	...	0	-2052.0	1	...
483	1	1	0	0	0	1	0	1	0	0	0	1	...	0	-2052.0	2	...
489	1	1	0	0	0	1	1	1	0	0	1	1	...	1	-2052.0	1	...
490	1	1	0	0	0	1	1	1	0	0	0	1	...	1	-2052.0	3	...
491	1	1	0	0	0	1	0	1	0	0	1	1	...	1	-2052.0	1	...
492	1	1	0	0	0	1	0	1	0	0	0	0	...	1	-2052.0	1	...
676	1	1	0	0	0	1	0	1	0	0	0	1	...	1	-2052.0	1	...
836	0	0	1	1	0	0	1	0	1	1	1	0	...	1	-2052.0	1	...
856	0	0	1	1	0	0	1	0	1	0	0	1	...	1	-2052.0	1	...
876	0	0	1	1	1	0	1	0	1	0	1	1	...	0	-2052.0	1	...
473	0	1	1	1	1	0	0	0	1	0	0	0	...	0	-1972.0	2	...
740	0	1	1	1	1	0	0	0	0	1	1	1	...	0	-1972.0	1	...
851	1	0	0	0	0	0	1	0	0	0	1	1	...	0	-1972.0	1	...
852	1	0	0	0	1	0	0	0	0	0	0	1	...	0	-1972.0	1	...
655	1	1	0	0	1	1	0	1	0	0	0	1	...	0	-1924.0	1	...
301	0	1	1	1	0	0	0	0	1	1	0	0	...	0	-1844.0	1	...
788	0	1	1	1	0	0	0	0	1	0	0	1	...	0	-1844.0	1	...
832	1	1	0	0	0	1	0	1	0	0	0	1	...	1	-1796.0	1	...
742	0	0	0	0	0	0	1	1	1	0	0	1	...	1	-1792.0	1	...
472	0	1	1	1	1	0	0	0	1	1	0	0	...	0	-1716.0	2	...
830	0	1	1	1	1	0	0	0	1	0	0	1	...	0	-1716.0	1	...
541	0	1	1	1	0	0	0	0	1	1	0	0	...	1	-1588.0	1	...

```
731   0   1   1   1   1   0   1   0   1   1   0   0 ...   0 -1588.0       1 ...
861   0   1   1   1   0   0   0   0   1   1   0   1 ...   1 -1588.0       1 ...
868   0   1   1   1   0   0   0   0   1   1   1   1 ...   0 -1332.0       1 ...
['BINARY', 884 rows, 2000 samples, 15 variables]
```

In [ ]:
