# Supplementary material for: HUBO and QUBO models for prime factorization
Source: Sci Rep. 2023 Jun 21;13:10080. doi: 10.1038/s41598-023-36813-x (PMC10284802; doi:10.1038/s41598-023-36813-x)
Supplement: Supplementary file 1 — Supplementary Information. [file 41598_2023_36813_MOESM1_ESM.zip › PF_RSA_100x_small.pdf]

In [1]:
```
####################################################
###       Prime factorization for RSA keys       ###
###        Code developer: Kyungtaek Jun         ###
###     Code version 1.0 - integer variable      ###
####################################################

##### Initial setting #####
# x1 = q0 + 2q1 + 4q2 + --- + 2^(n-1)q(n-1)
# x2 = qn + 2q(n+1) + 4q(n+2) + ---  2^(n-1)q(2n-1)
# x1x2 = c

### Least square problem
# QUBO = (x1x2 - c)^2 - c^2

import numpy as np
import random, math
import copy
from dwave.system import DWaveSampler, EmbeddingComposite
import dimod


x1 = int(10111)
x2 = int(101)
c = x1*x2
print ("first prime number: ",x1)
print ("second prime number: ",x2)
print ("RSA number: ",c)
```

```
first prime number:  10111
second prime number:  101
RSA number:  1021211
```

In [2]:
```
qubits = 14
max_d = format(len(str(2*qubits)), '02')
QM = np.zeros((2*qubits, 2*qubits))
Q = {}

for j in range(qubits):
    for i in range(qubits):
        po1 = i
        po2 = qubits+j
        val = pow(2,2*(i+j)) - 2*c*pow(2,i+j)
```

```
        exec("Q.update({('q%s','q%s'):%s})"%(format(po1+1,max_d),format(po2+1,max_d), format(val)))

for k in range(qubits):
    for i in range(qubits-1):
        for j in range(i+1,qubits):
            #2^(i+j+2k+1)aiajbk
            po1 = i
            po2 = j
            po3 = qubits+k
            val = pow(2,i+j+2*k+1)
            exec("Q.update({('q%s','q%s','q%s'):%s})"%(format(po1+1,max_d),format(po2+1,max_d),
                                                   format(po3+1,max_d), format(val)))

for k in range(qubits):
    for i in range(qubits-1):
        for j in range(i+1,qubits):
            #2^(i+j+2k+1)akbibj
            po1 = k
            po2 = qubits+i
            po3 = qubits+j
            val = pow(2,i+j+2*k+1)
            exec("Q.update({('q%s','q%s','q%s'):%s})"%(format(po1+1,max_d),format(po2+1,max_d),
                                                   format(po3+1,max_d), format(val)))

for i2 in range(qubits-1):
    for j2 in range(i2+1,qubits):
        for i1 in range(qubits-1):
            for j1 in range(i1+1,qubits):
                po1 = i1
                po2 = j1
                po3 = qubits+i2
                po4 = qubits+j2
                val = pow(2,i1+j1+i2+j2+2)
                exec("Q.update({('q%s','q%s','q%s','q%s'):%s})"%(format(po1+1,max_d),format(po2+1,max_d),
                                                            format(po3+1,max_d), format(po4+1,max_d), format(val)))
```

In [3]:

```
sampler_auto = EmbeddingComposite(DWaveSampler(solver={'qpu': True}))
sampleset = dimod.ExactPolySolver().sample_hubo(Q)

# energy = 0
energies = sampleset.record.energy
energy0_nums = np.where(energies==-pow(c,2))[0]
x = np.zeros(2)
```

```
for idx in range(len(energy0_nums)):
    sol1 = sampleset.record[energy0_nums[idx]][0]
    for xk in range(2):
        x[xk]=0
    lambda1 = 0
    for xk in range(2):
        for k in range(qubits):
            x[xk] = x[xk] + pow(2,k)*(sol1[xk*qubits+k])

    print(x)
```

```
[10111.   101.]
[  101. 10111.]
```

In [4]:
```
print(sampleset.first)
```

```
Sample(sample={'q01': 1, 'q02': 1, 'q03': 1, 'q04': 1, 'q05': 1, 'q06': 1, 'q07': 1, 'q08': 0, 'q09': 1, 'q10': 1, 'q11': 1, 'q12': 0, 'q13': 0, 'q14': 1, 'q15': 1, 'q16': 0, 'q17': 1, 'q18': 0, 'q19': 0, 'q20': 1, 'q21': 1, 'q22': 0, 'q23': 0, 'q24': 0, 'q25': 0, 'q26': 0, 'q27': 0, 'q28': 0}, energy=-1042871906521.0, num_occurrences=1)
```
