# Supplementary material for: HUBO and QUBO models for prime factorization
Source: Sci Rep. 2023 Jun 21;13:10080. doi: 10.1038/s41598-023-36813-x (PMC10284802; doi:10.1038/s41598-023-36813-x)
Supplement: Supplementary file 1 — Supplementary Information. [file 41598_2023_36813_MOESM1_ESM.zip › PF_RSA_Subrange.pdf]

In [91]:
```
##### Initial setting #####
# p = q0 + 2q1 + 4q2 + --- + 2^(n-1)q(n-1)
# q = qn + 2q(n+1) + 4q(n+2) + ---  2^(n-1)q(2n-1)
# pq = N

### Least square problem
# HUBO = (pq - N)^2 - N^2

import numpy as np
import random, math
import copy
from dwave.system import DWaveSampler, EmbeddingComposite
import dimod
from decimal import Decimal

p = int(1000033)
q = int(1000037)
N = p*q
print ("first prime number: ",p)
print ("second prime number: ",q)
print ("RSA number: ",N)
```

```
first prime number:  1000033
second prime number:  1000037
RSA number:  1000070001221
```

In [92]:
```
Si = int(1000000)
Sj = int(1000000)
qubits = 6
max_d = format(len(str(2*qubits)), '02')
QM = np.zeros((2*qubits, 2*qubits))
Q = {}
GME = -N*N - Si*Si*Sj*Sj + 2*N*Si*Sj
print("Required global minimum energy: ", GME)
# linear subrange terms
for k in range(qubits):
    po = k
    val = (pow(2,2*k) + pow(2,k+1)*Si)*Sj*Sj - 2*N*Sj*pow(2,k)
    exec("Q.update({('q%s','q%s'):%s})"%(format(po+1,max_d),format(po+1,max_d), format(val)))

for k in range(qubits):
    po = k + qubits
```

```
    val = (pow(2,2*k) + pow(2,k+1)*Sj)*Si*Si - 2*N*Si*pow(2,k)
    exec("Q.update({('q%s','q%s'):%s})"%(format(po+1,max_d),format(po+1,max_d), format(val)))

# quadratic subrange terms
for i in range(qubits-1):
    for j in range(i+1,qubits):
        po1 = i
        po2 = j
        val = Sj*Sj*pow(2,i+j+1)
        exec("Q.update({('q%s','q%s'):%s})"%(format(po1+1,max_d),format(po2+1,max_d), format(val)))

for i in range(qubits-1):
    for j in range(i+1,qubits):
        po1 = i + qubits
        po2 = j + qubits
        val = Si*Si*pow(2,i+j+1)
        exec("Q.update({('q%s','q%s'):%s})"%(format(po1+1,max_d),format(po2+1,max_d), format(val)))

for i in range(qubits):
    for j in range(qubits):
        po1 = i
        po2 = j + qubits
        val = pow(2,2*(i+j)) + pow(2,i+2*j+1)*Si + pow(2,2*i+j+1)*Sj + Si*Si*pow(2,i+j+2) - N*pow(2,i+j+1)
        exec("Q.update({('q%s','q%s'):%s})"%(format(po1+1,max_d),format(po2+1,max_d), format(val)))

# cubic subrange terms
for k in range(qubits):
    for i in range(qubits-1):
        for j in range(i+1,qubits):
            #2^(i+j+2k+1)aiajbk
            po1 = i
            po2 = j
            po3 = qubits+k
            val = pow(2,i+j+1)*(pow(2,2*k) + pow(2,k+1)*Sj)
            exec("Q.update({('q%s','q%s','q%s'):%s})"%(format(po1+1,max_d),format(po2+1,max_d),
                                                        format(po3+1,max_d), format(val)))

for k in range(qubits):
    for i in range(qubits-1):
        for j in range(i+1,qubits):
            #2^(i+j+2k+1)akbibj
            po1 = k
            po2 = qubits+i
            po3 = qubits+j
```

```
            val = pow(2,i+j+1)*(pow(2,2*k) + pow(2,k+1)*Si)
            exec("Q.update({('q%s','q%s','q%s'):%s})"%(format(po1+1,max_d),format(po2+1,max_d),
                                                       format(po3+1,max_d), format(val)))
# quartic subrange terms
for i2 in range(qubits-1):
    for j2 in range(i2+1,qubits):
        for i1 in range(qubits-1):
            for j1 in range(i1+1,qubits):
                po1 = i1
                po2 = j1
                po3 = qubits+i2
                po4 = qubits+j2
                val = pow(2,i1+j1+i2+j2+2)
                exec("Q.update({('q%s','q%s','q%s','q%s'):%s})"%(format(po1+1,max_d),format(po2+1,max_d),
                                                                 format(po3+1,max_d), format(po4+1,max_d), format(val)))
```

```
Required global minimum energy:  -4900170941490841
```

In [93]:

```
sampler_auto = EmbeddingComposite(DWaveSampler(solver={'qpu': True}))
sampleset = dimod.ExactPolySolver().sample_hubo(Q)

# energy = 0
energies = sampleset.record.energy
energy0_nums = np.where(energies==GME)[0]
x = np.zeros(2)
for idx in range(len(energy0_nums)):
    sol1 = sampleset.record[energy0_nums[idx]][0]
    for xk in range(2):
        x[xk]=0
    lambda1 = 0
    for xk in range(2):
        for k in range(qubits):
            x[xk] = x[xk] + pow(2,k)*(sol1[xk*qubits+k])

    print(x)
print ("prime number: ",x+[Si,Sj])
```

```
[37. 33.]
[33. 37.]
prime number:  [1000033. 1000037.]
```

In [ ]:
