# Supplementary material for: HUBO and QUBO models for prime factorization
Source: Sci Rep. 2023 Jun 21;13:10080. doi: 10.1038/s41598-023-36813-x (PMC10284802; doi:10.1038/s41598-023-36813-x)
Supplement: Supplementary file 1 — Supplementary Information. [file 41598_2023_36813_MOESM1_ESM.zip › Supplementary_Information.docx]

HUBO and QUBO models for prime factorization

Kyungtaek Jun^1,2,*^, Hyunju Lee^3^

1. German Engineering Research and Development Center, LSTME Busan Branch, South Korea
2. Institute of Mathematical Sciences, Ewha Womans University, South Korea
3. University-Industry Foundation, Yonsei University Health System, South Korea

∗Corresponding author: ktfriends@gmail.com

We provide Python codes used in the manuscript. To test these codes, simply change the given prime number.

Code1. “PF_QUBO. PDF” This code uses a quantum annealer by changing the HUBO model to the QUBO model for a prime factorization model.

Code2. “PF_RSA_14qubits.PDF” This code is the result of using the quantum annealer for the HUBO model of a prime factorization model.

Code3. “PF_RSA_Subrange.PDF” This code is the result of using the quantum annealer by applying the range dependent Hamiltonian algorithm to the HUBO model for a prime factorization model.

Code4. “PF_RSA_100x_small.PDF” This code is the result of a prime factorization of numbers where the magnitudes of the two primes differ by about 100 times.
